# Encoding and decoding selectivity and promiscuity in the human chemokine-GPCR interaction network

**DOI:** 10.1016/j.cell.2025.03.046

**Published:** 2025-04-23

**Authors:** Andrew B. Kleist, Martyna Szpakowska, Lindsay J. Talbot, Greg Slodkowicz, Duccio Malinverni, Monica A. Thomas, Kyler S. Crawford, Daniel J. McGrail, Acacia F. Dishman, Michael J. Wedemeyer, Madison Sluter, S. Stephen Yi, Nidhi Sahni, Francis C. Peterson, Andy Chevigné, Brian F. Volkman, M. Madan Babu

**Affiliations:** 1 Department of Biochemistry, Medical College of Wisconsin, Milwaukee, WI, USA; 2 Medical Scientist Training Program, Medical College of Wisconsin, Milwaukee, WI, USA; 3 MRC Laboratory of Molecular Biology, Cambridge, UK; 4 Immuno-Pharmacology and Interactomics, Department of Infection and Immunity, Luxembourg Institute of Health, Esch-sur-Alzette, Luxembourg; 5 Department of Bone Marrow Transplantation and Cellular Therapy, St. Jude Children’s Research Hospital, Memphis, TN, USA; 6 Department of Surgery, St. Jude Children’s Research Hospital, Memphis, TN, USA; 7 Center of Excellence for Data-Driven Discovery, Department of Structural Biology, St Jude Children’s Research Hospital, Memphis, TN, USA; 8 Department of Systems Biology, University of Texas MD Anderson Cancer Center, Houston, TX, USA; 9 Department of Oncology, Dell Medical School, University of Texas at Austin, Austin, TX, USA; 10 Department of Biomedical Engineering, Cockrell School of Engineering, University of Texas at Austin, Austin, TX, USA; 11 Department of Bioinformatics and Computational Biology, University of Texas MD Anderson Cancer Center, Houston, TX, USA; 12 Program in Quantitative and Computational Biosciences, Baylor College of Medicine, Houston, TX, USA; 13 Department of Epigenetics & Molecular Carcinogenesis, University of Texas MD Anderson Cancer Center, Houston, TX, USA; 14 Protein Foundry, LLC, West Allis, WI, USA; 15 Program in Chemical Biology, Medical College of Wisconsin, Milwaukee, WI, USA; 16 Mellowes Center for Genomic Sciences and Precision Medicine, Medical College of Wisconsin, Milwaukee, WI, USA; 17 Present address: Center of Excellence for Data-Driven Discovery, Department of Structural Biology, St. Jude Children’s Research Hospital, Memphis, TN, USA; 18 Lead contact

## Abstract

In humans, selective and promiscuous interactions between 46 secreted chemokine ligands and 23 cell surface chemokine receptors of the G-protein-coupled receptor (GPCR) family form a complex network to coordinate cell migration. While chemokines and their GPCRs each share common structural scaffolds, the molecular principles driving selectivity and promiscuity remain elusive. Here, we identify conserved, semi-conserved, and variable determinants (i.e., recognition elements) that are encoded and decoded by chemokines and their receptors to mediate interactions. Selectivity and promiscuity emerge from an ensemble of generalized (“public/conserved”) and specific (“private/variable”) determinants distributed among structured and unstructured protein regions, with ligands and receptors recognizing these determinants combinatorially. We employ these principles to engineer a viral chemokine with altered GPCR coupling preferences and provide a web resource to facilitate sequence-structure-function studies and protein design efforts for developing immuno-therapeutics and cell therapies.

## In brief

Sequence and structure-based analysis highlight the molecular principles driving chemokine-GPCR selectivity and promiscuity, enabling the design of chemokines with altered receptor preferences.

## Graphical abstract

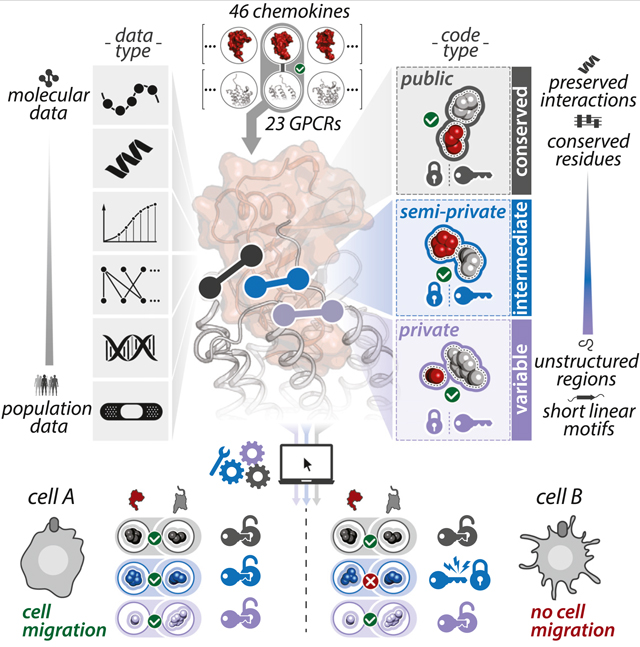

## INTRODUCTION

**Figure 1.**
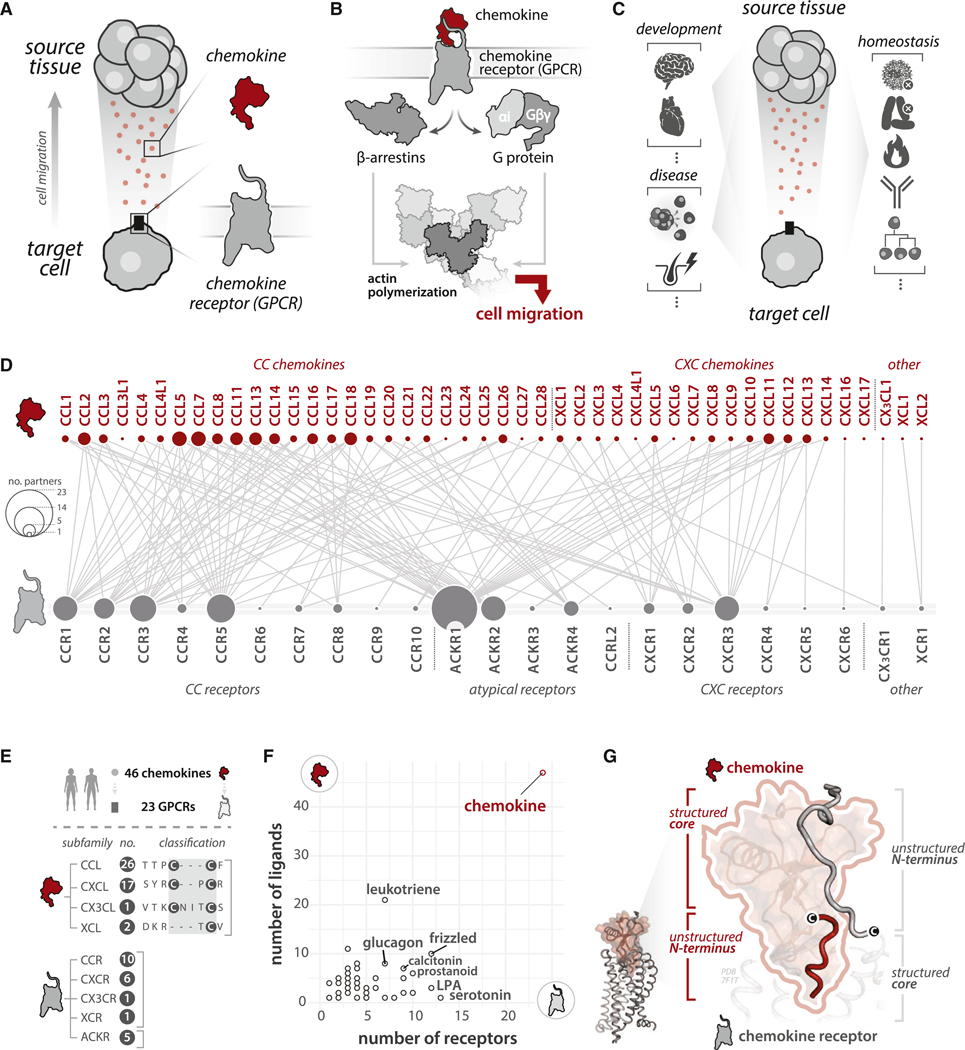
Structural and functional organization of the chemokine-GPCR system (A) Secreted chemokines are sensed by receptors on migrating cells. (B) Chemokine binding to GPCRs activates G protein and β-arrestin signaling/recruitment, resulting in cell migration. (C) The chemokine-GPCR system underlies development and immune regulation and is exploited in disease. (D) Interaction network between chemokine ligands (top) and receptors (bottom) (compiled from Table S1; interaction strength ≥ 2 represented, see STAR Methods). Node size scaled to number of binding partners. (E) Chemokine ligand (red) and receptor (gray) subfamilies. (F) The number of GPCR ligands and receptors grouped by family (STAR Methods). (G) Reciprocal structured-to-unstructured binding mode (PDB: 7F1T). The receptor’s unstructured N terminus interacts with the chemokine’s structured core, and vice versa. See also Figure S1.

In humans, cell migration is coordinated by interactions between secreted chemokine ligands and their chemokine receptors of the G-protein-coupled receptors (GPCRs) superfamily that are expressed on the surface of the migrating cell (Figure 1A). Chemokine-GPCR interactions initiate signaling cascades through G protein^1,2^ and β-arrestin^3^ pathways, resulting in directed migration of the target cell along a chemokine gradient (Figure 1B). In this manner, a combination of chemokines secreted by diverse cell types is interpreted by receptors on receiving cells to coordinate a vast cell migration system throughout the body. This ligand-receptor system forms the molecular basis for cell migration in developmental and homeostatic processes, including cerebellar development,^4,5^ gastrointestinal vascularization,^6^ coronary artery development,^7^ hematopoiesis,^8,9^ and immunity^10,11^ (Figure 1C). This system is exploited in disease processes including cancer metastasis,^12–15^ autoimmune diseases,^16^ bleeding disorders,^17^ COVID-19 pathogenicity,^18–20^ HIV infection,^21,22^ and viral immune evasion^23^ (Figure 1C).

The diverse roles of the chemokine-GPCR system are enabled by a complex network of interactions among 46 chemokine ligands and 23 receptors (Figures 1D and 1E), which together comprise the most abundant genetically encoded ligand-receptor system among the GPCR superfamily (Figure 1F). A comprehensive literature survey allowed us to construct a network >100 experimentally validated chemokine-GPCR interactions highlighting selective (i.e., non-overlapping) and promiscuous (i.e., overlapping) interactions in the network (Figure 1E; Figure S1; Table S1). Spatial (e.g., cell- and tissue-specific) and temporal regulation of chemokine and GPCR expression patterns further enable this system to coordinate an extensive cell migration network throughout the body with remarkable precision. In effect, chemokines function as molecular signposts, controlling the flow of cellular traffic throughout the body via selective GPCR interactions.

Despite its complexity, this system is constructed using conserved chemokine and GPCR structural folds that interact in a prescribed manner^24^: the receptor’s unstructured N terminus interacts with the chemokine’s structured core and vice versa (Figure 1G). More than a dozen chemokine-GPCR complex structures have demonstrated this reciprocal structured-to-unstructured pairing. Within this framework, additional mechanisms allow chemokines and GPCRs to interact with some binding partners and not others. Uncovering the determinants and how they are encoded can reveal the molecular basis of selectivity and promiscuity in this critical ligand-receptor system and accelerate design of chemokine-based therapeutics.

In this study, we develop a data science framework, integrating diverse data types to uncover molecular principles governing the emergence of a complex interaction network from two highly conserved protein architectures. We used the identified patterns to engineer chemokine mutants with altered receptor binding profiles, providing evidence that these principles can be leveraged to design selective chemokine-GPCR interactions. Finally, we present sequence conservation, genome variation, and disease mutation data, and literature-validated chemokine-GPCR interactions in a comprehensive online resource for the scientific community (https://andrewbkleist.github.io/chemokine_gpcr_encoding/).

## RESULTS

### Common numbering and sequence alignments

Functionally important residues can be identified by analyzing structurally equivalent positions from protein sequence alignments.^25^ We identified 46 human chemokine paralogs and their respective one-to-one orthologs from over 60 species (STAR Methods) to construct a master alignment of 1,058 sequences. Using this alignment and 35 available chemokine structures, we devised a common chemokine numbering (CCN) system to annotate structurally equivalent positions for all members of the chemokine family (Figures S2A–S2C). Each position is defined by two attributes (SSE.P): SSE refers to the consensus secondary structure element (SSE) that we defined using the available structures (Figure S2B), and P refers to the position (P) within the consensus SSE. For instance, the last of the four conserved cysteines, residue 50 in CXCL12 and residue 48 in CCL2, are denoted as B3.3 (third position in the third b strand) (Figure S2A). Loops are denoted in lowercase by the SSEs they connect; thus, b1b2.1 refers to the first position in the loop connecting β1 and β2 strands.

We implemented the same approach for chemokine GPCRs, by constructing an alignment of 951 chemokine-receptor sequences, including all 23 human paralogs and one-to-one orthologs from over 60 species (STAR Methods). Structurally equivalent sequence position names were mapped using a modified version of the GPCR database (GPCRdb) numbering system,^26^ termed chemokine-receptor numbering (CRN, STAR Methods), which considers a conserved N-terminal cysteine among chemokine GPCRs.^27^

Using CCN and CRN alignments (Figure S2C), we calculated sequence conservation scores for every chemokine and GPCR position among (1) human chemokine and receptor paralogs, i.e., conservation among *different* (*paralogous*) chemokines/GPCRs within the *same* species (humans); and (2) chemokine and receptor orthologs, i.e., conservation of the *equivalent* (*orthologous*) chemokine/GPCR among *different* species (STAR Methods). Only conventional (i.e., non-atypical) chemokine receptors were used to score paralog conservation.

### Constrained plasticity in chemokine-receptor-binding mode is mediated by a minimal network of conserved residues

We gathered 14 published chemokine-GPCR complex structures and two validated models available at the time^28–41^ and assigned CCN and CRN for the residues within each complex (Figure S3A). Despite high structural similarity of chemokines and their GPCRs, we observed a range in the orientation of chemokine-GPCR complexes, with some pairwise root-mean-square deviation (RMSD) values varying from 17 to <1 Å (Figure S3B). We used paired GPCR coordinates to perform pairwise structural alignments of all complexes, then calculated pairwise Cα RMSD values for structurally equivalent chemokine positions. While some chemokine regions exhibit a high degree of structural plasticity, others are more constrained, with a mean RMSD of ~5 Å or less, with low-RMSD positions occurring at or near disulfide-bonded cysteines (Figure S3C).

**Figure 2.**
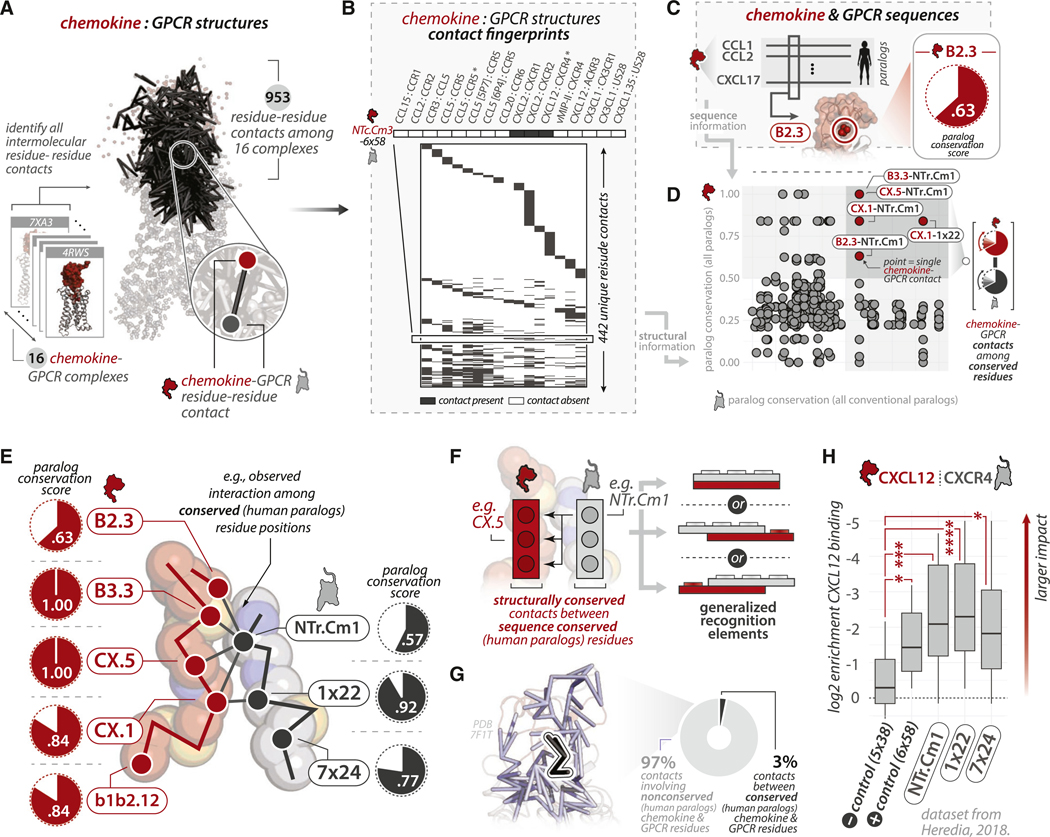
Minimal encoding of generalized chemokine-GPCR recognition (A) Residue-residue contacts for 16 chemokine-GPCR complexes. (B) Contact fingerprint representing all unique contacts (rows) and complexes (columns). Example fingerprint shown with black/white denoting presence/absence of contact between equivalent residues. * denotes two models used. (C) Human paralog alignments were used to calculate paralog conservation scores, with CCN example given for position B2.3. (D) Residue-residue contacts among 16 chemokine-GPCR complexes (points), and human paralog conservation scores of chemokine (y axis) and receptor (x axis) residues comprising each contact. Receptor paralog scores are calculated among non-atypical receptors (STAR Methods). (E) 12/16 complexes have ≥1 contact between conserved chemokine/receptor residues (human paralogs) involving disulfide regions. Paralog conservation scores shown as pie charts. (F) Interactions between paralog conserved residues by analogy to Lego bricks. (G) Contacts between conserved (human paralogs) chemokine and GPCR residues (dark gray) are 3% of overall contacts. Contacts involving ≥1 variable residue are 97% of contacts (light blue). (H) Effects of CXCR4 mutagenesis on CXCL12 binding. Log_2_ enrichment scores reflect CXCL12-GFP binding to cells harboring WT versus mutated CXCR4 (STAR Methods). Statistical testing by Kruskal-Wallis test with *p* values determined by post hoc Dunn test with Bonferroni correction for multiple comparisons. *p value < 0.05, ***p value < 0.001, ****p value < 0.0001. *p* values for all other pairwise comparisons > 0.05. Boxplot boxes reflect the median (central line), first and third quartiles (box boundaries), and largest/smallest values no further than 1.5x the interquartile range (whiskers). Raw data from Heredia et al.^43^ See also Figures S2 and S3.

The preserved positioning of some structural elements suggests that broadly preserved molecular contacts may constrain chemokine-GPCR interactions. We use the term “preservation” to refer to the degree of variability of features among different sets of chemokine-GPCR *structures* (and in contrast to “conservation,” which we use to refer to variability among sets of *sequences*). To identify those contacts, we calculated all non-covalent, intermolecular residue-residue contacts for all 16 complexes (Figure 2A; Figure S3D).^42^ We identified 953 total contacts, 442 of which occurred between unique pairs of chemokine and GPCR positions (Figure 2B). Mapping paralog conservation scores to intermolecular chemokine-GPCR contacts (Figure 2C) revealed only 5 contacts were formed between conserved chemokine and GPCR residues (Figure 2D), with at least one such contact being present in a majority of complexes (12/16).

Among the 5 conserved chemokine and GPCR residues participating in these contacts, all but one *participate in* or are *adjacent to* a disulfide bond (Figure 2E). Given the essential functional role of receptor transmembrane domain (TM)1–TM7 disulfides,^44^ it is likely that the interactions between conserved, disulfide-rich regions in the respective binding partners facilitate the selection of their cognate chemokine receptors from other class A GPCRs; most of which lack a TM1–TM7 disulfide.^27^ In effect, chemokine disulfides are repurposed for molecular recognition of cognate chemokine GPCRs, thereby maximizing the functional utility of a minimal set of residues. Notably, none of the generalized recognition contacts are structurally preserved among *all* complexes despite the constituent residues being highly conserved in sequence. Instead of using a single, structurally preserved constraint, chemokine-GPCR interactions employ a series of soft constraints that can be mixed and matched along different registers, allowing pair-specific local rearrangements. This is analogous to two Lego bricks that can be connected to one another in different registers using conserved “knobs” and “holes” (Figure 2F). In turn, the degeneracy of these local constraints may facilitate the variation in chemokine orientation observed in chemokine-GPCR complexes.

We next assessed the extent to which these contacts contribute to the total interface. Pairwise contacts among paralog conserved residues account for only 3% of the total contacts, whereas the remaining contacts involve at least one poorly conserved residue (i.e., paralog conservation scores < 0.5; Figure 2G). These data reveal that generalized recognition between chemokine and GPCR binding partners is encoded at a single interface region using a minimal set of residues, thereby maximizing the surface area dedicated to pair-specific recognition elements.

How important are the residues comprising disulfide-rich hotspots in chemokine-GPCR binding? A recent study^43^ assessed the effects of CXCR4 mutations on CXCL12 binding via saturation mutagenesis (i.e., substitution of every possible amino acid at every position of CXCR4). Using this dataset, we compared the effects of CXCR4 mutations at conserved hotspot residues (Pro27^NTr.Cm1^, Cys28^1×22^, and Cys274^7x24^) with those at other positions that are either (1) known to be important for CXCL12 binding (e.g., Asp262^6x58^; positive control) or (2) non-interface positions in the vicinity of the binding pocket that are likely to be unimportant for binding (e.g., Val198^5x38^; negative control; STAR Methods). Mutations of receptor residues involved in or adjacent to contacts among conserved chemokine- and GPCR residues (i.e., NTr.Cm1, 1×22, and 7×24) significantly reduce CXCL12 binding (Figure 2H), consistent with alanine mutagenesis studies of CCR8 (i.e., Cys25Ala^1×22^ or Cys272Ala^7×24^)^44^ and CXCR4 (Cys28Ser^1×22^ and Cys274Ser^7×24^).^45^

### Semi-preserved molecular “sensors” guide subfamily-specific recognition

**Figure 3.**
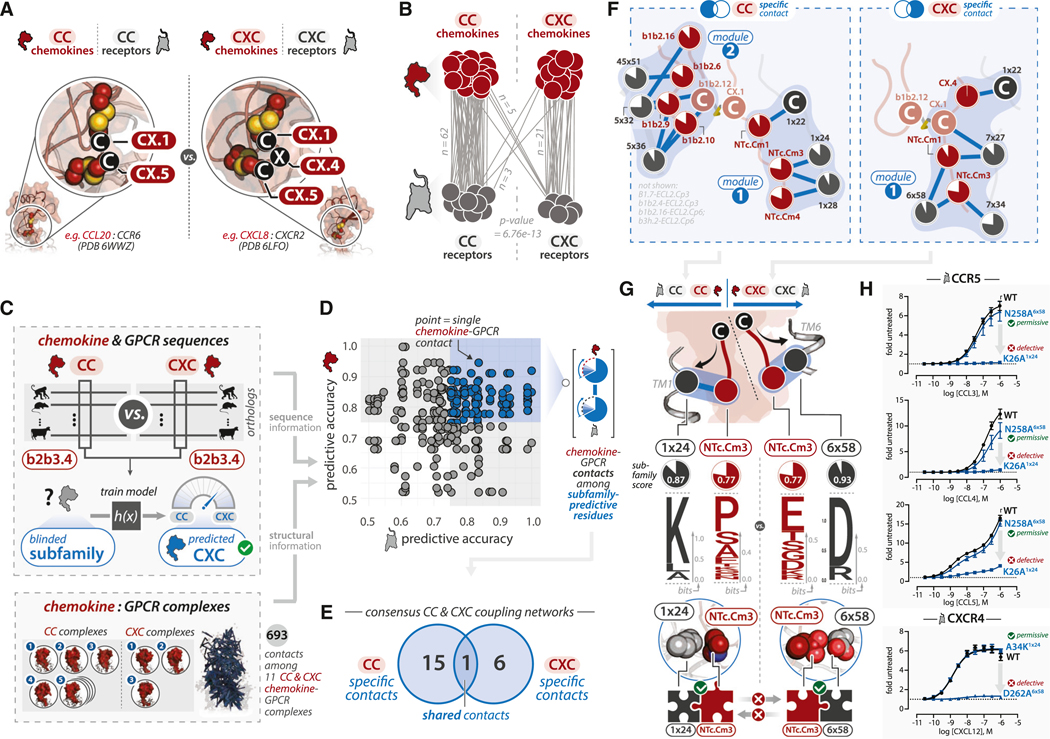
Subfamily-specific sensors encode distinct chemokine-GPCR binding modes (A) CC and CXC chemokines differ by the presence (CXC) or absence (CC) of a residue (“X”) between conserved N-terminal cysteines. (B) Predominance of interactions among like subfamily chemokines/GPCRs (chi-squared test *p* = 6.76e-11). (C) Top: logistic regression models were trained to classify a sequence as CC or CXC by residue identity at each sequence position. Positions ranked according to accuracy in subfamily prediction (STAR Methods). Bottom: CC and CXC complexes used to identify consensus subfamily contacts. Contacts in CCL5:CCR5 complexes were considered degenerate (STAR Methods). (D) Residue-residue contacts among CC and CXC complexes (points), with subfamily-predictive scores of chemokine (y axis) and receptor (x axis) positions comprising each contact. (E) Consensus, CC- and CXC-specific contacts involving subfamily-predictive positions. (F) CC- and CXC-specific consensus contacts from (E), with residue positions (chemokine: red, receptor: gray) represented as pie charts depicting position-specific subfamily scores. (G) CC- and CXC-specific consensus contact (top) with subfamily scores and residue sequence logos (middle). CC and CXC complexes (bottom) with puzzle pieces depicting how the same chemokine position (NTc.Cm3) uniquely accommodates CC versus CXC receptor residues (bottom). (H) β-arrestin recruitment by NanoLuc Binary Technology (NanoBiT) with CCR5 (top 3 panels; versus CCL3, CCL4, and CCL5) and CXCR4 (bottom panel; versus CXCL12) receptor mutants at positions 1×24 and 6×58. All experiments *n* = 3. Error bars reflect SEM. See Table S2. See also Figure S4.

A majority of human chemokines belong to either CC (26 members) or CXC (17 members) subfamilies, named for the presence (i.e., CXC) or absence (i.e., CC) of a single residue (i.e., the “X” in CXC: CCN position CX.4) between the two conserved N-terminal cysteines (Figure 3A). This difference has significant functional consequences, as CC and CXC chemokines predominantly couple to CC or CXC receptors, respectively (Figure 3B). To identify whether positions other than the “X” might confer sub-family-specific recognition, we devised a logistic regression classification algorithm to evaluate the predictive accuracy of other CCN positions at discriminating a chemokine as belonging to CC or CXC subfamilies (Figure 3C, top; STAR Methods). Resulting “subfamily scores,” calculated for each CCN position, reflect the algorithm’s accuracy at predicting the identity (i.e., CC versus CXC) of an unknown sequence. As anticipated, the “X” residue (CCN: CX.4) was the most predictive of chemokine subfamily, but unexpectedly, we identified 34 other positions at the binding interface that are predictive with an accuracy ≥ 75% (Figure S4A). Subfamily-predictive positions were distributed across almost every chemokine SSE, suggesting that subfamily-specific differences are widespread beyond the “X” residue. Of note, residue conservation of subfamily-predictive positions among CC and CXC chemokines was variable, with some subfamily-predictive positions having high paralog conservation in either or both subfamilies (but different amino acids); whereas other subfamily-predictive positions showed low paralog conservation among CC and CXC subfamilies (Figure S4B). The latter positions are predictive despite low paralog conservation because CC and CXC chemokines employ different *sets* of amino acids even if no specific amino acid is dominant among either subfamily (Figure S4C).

While chemokine subfamilies are classified by the presence or absence of a residue between two N-terminal cysteines, CC versus CXC receptor classification is based on the subfamily of ligands with which they primarily interact. To identify whether chemokine receptors possess subfamily discriminating positions, we applied the classifier approach to receptor alignments (Figure 3C, top; STAR Methods). We identified 29 CRN positions at the binding interface that were ≥75% accurate in discriminating CC versus CXC receptors (Figure S4A). Some subfamily-predictive positions were broadly conserved, while others displayed low paralog conservation among both subfamilies (Figure S4B). As with ligands, subfamily-predictive positions that have poor conservation among CC and CXC receptor paralogs employ different sets of amino acids among CC and CXC subfamilies (Figure S4C). These differences might enable receptors to customize the level of specificity and promiscuity among individual chemokine-GPCR pairs within a particular subfamily.

How often do subfamily-predictive positions interact with one another in chemokine-GPCR complexes? For all intermolecular residue contacts among complexes made of “like” CC or CXC ligand-receptor pairs (Figure 3C, bottom), we mapped subfamily scores for the chemokine and GPCR residues comprising each contact (Figure 3D). We identified 106 contacts between distinct pairs of chemokine and GPCR positions that have ≥75% accuracy at predicting the respective chemokine or GPCR subfamily. Of these, we identified those present in a majority of CC but not CXC complexes and vice versa (STAR Methods). We considered contacts between subfamily-predictive positions in one binding partner and conserved positions in the other since the subfamily-predictive positions could cause distinct, subfamily-specific modes of interaction with conserved residues. These analyses revealed 15 (CC) and 6 (CXC) contacts involving subfamily-specific residue positions found exclusively in chemokine-GPCR complexes of the respective subfamilies (Figures 3E and 3F).

Comparison of CC- and CXC-specific residue contact networks revealed that the same, conserved receptor position (Cys^1×22^) is contacted differently by CC and CXC chemokines. CC chemokines contact this residue using position NTc.Cm1, whereas CXC chemokines contact this residue using the “X” position CX.4, both of which are subfamily-predictive positions (Figures 3F and S4A). This subtle structural alteration may function to derivatize the conserved architecture to allow more pronounced differences in interactions in the binding pocket.^39,46^ In the receptor binding pocket, CC chemokines use position NTc.Cm3 to contact GPCR position 1×24, whereas CXC chemokines use NTc.Cm3 to contact 6×58 (Figure 3G). CC-subfamily GPCRs have a conserved positively charged Lys at position 1×24, whereas CXC subfamily GPCRs have a conserved negatively charged Asp at position 6×58. While the chemokine position NTc.Cm3 contacts these two GPCR positions in CC and CXC complexes, respectively, its residue identity is not strongly conserved among CC or CXC chemokines. Subfamily residues exhibit distinct identities, with Pro^NTc.Cm3^ being predominantly represented among CC chemokines, and Glu^NTc.Cm3^ being predominant represented among CXC chemokines (Figure 3G).

Together, these data suggest that subfamily specificity is enabled using reciprocal, subfamily-specific sensors on the chemokine and GPCR. To test this, we performed paired Ala mutagenesis of GPCR residues predicted to be important for CC versus CXC recognition using representative CC (i.e., CCR5) and CXC (i.e., CXCR4) receptors (Figure 3H; Table S2). At CCR5, Asn258Ala^6x58^ had negligible effects on β-arrestin recruitment compared with wild-type (WT) CCR5 in response to ligands CCL3, CCL4, and CCL5, whereas Lys26Ala^1×24^ caused near-complete loss of β-arrestin recruitment in response to all ligands even at high concentrations (Figure 3H). Conversely, at CXCR4, Asp262Ala^6x58^ resulted in a complete loss of β-arrestin recruitment in response to CXCL12, consistent with prior radioligand results,^47^ whereas Ala34Lys^1×24^ had no appreciable effect on β-arrestin recruitment compared with WT CXCR4. These data support a model in which chemokines and their GPCRs possess an intrinsic “handedness” that leads to differences in binding pocket targeting; this enables reciprocal recognition of subfamily-specific binding partners by employing subfamily-specific determinants.

### Customization of selectivity preferences among chemokine-GPCR complexes

**Figure 4.**
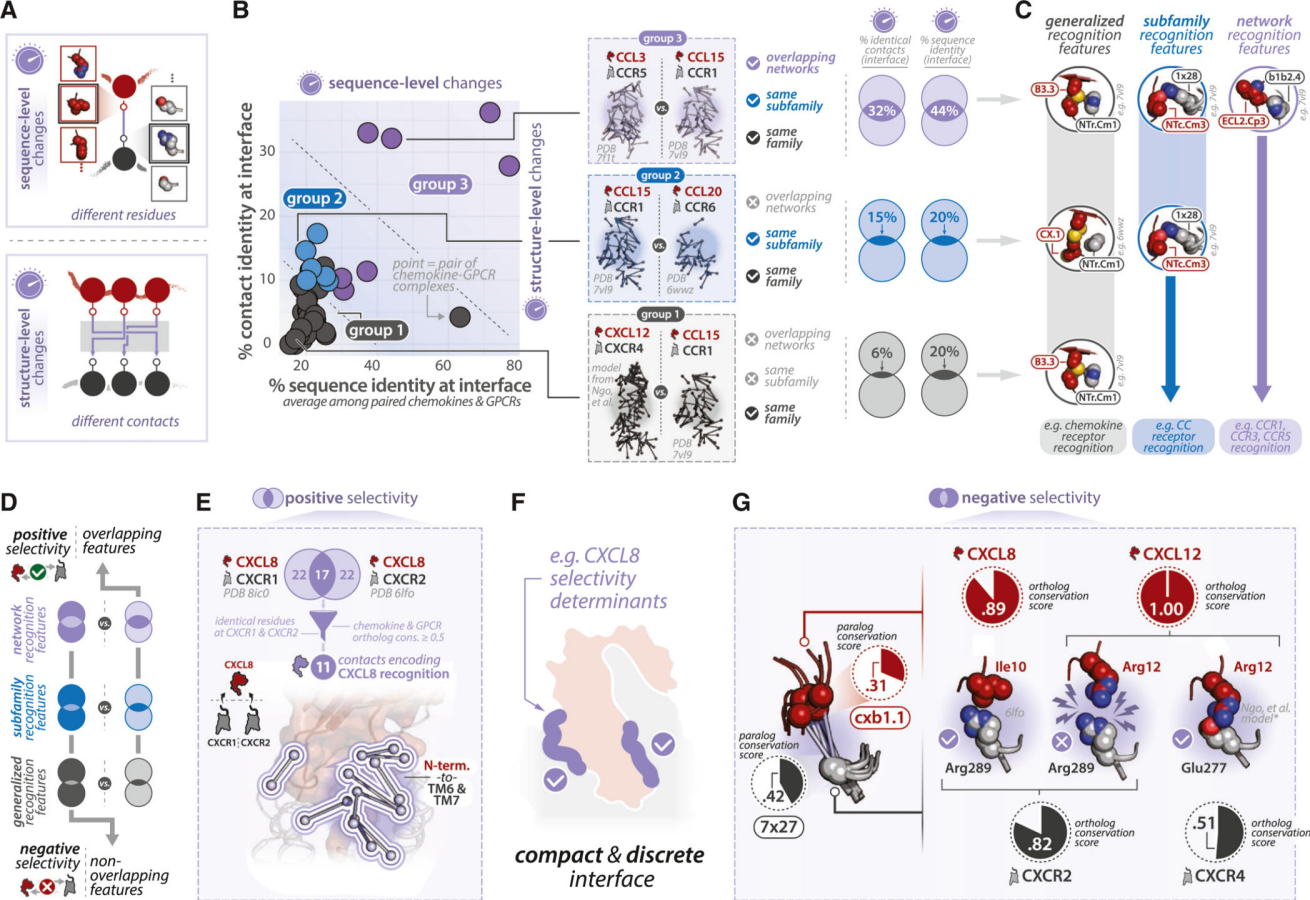
Customization of chemokine-GPCR interactions through structure- and sequence-level changes (A) Customization through structure-level (contact differences) and sequence-level (residue differences among structurally preserved contacts) changes. (B) Comparison of the *percent* identical/equivalent contacts at the interface among all chemokine-GPCR complexes (y axis, related to structure-level changes) and mean pairwise percent identities of interface residues among paired chemokines and GPCRs (x axis, related to sequence-level changes). Points denote a comparison of a pair of chemokine-GPCR complexes; examples given for the three groups of selectivity-network relatedness. (C) Generalized (i.e., distinguish chemokines/receptors from other molecules/GPCRs), subfamily (i.e., distinguish between chemokines/receptors of different subfamilies, CC or CXC), and network (i.e., found only within specific chemokine-receptor pairs that share interactions) recognition features. (D) Positive selectivity features facilitate interactions, and negative selectivity features disfavor interactions at generalized, subfamily, and network “layers” of encoding. (E) Positive selectivity example. 11 shared contacts among identical residues encode CXCL8 recognition by CXCR1 and CXCR2 (STAR Methods). (F) Selectivity preferences are encoded using compact, discrete interface regions. (G) Negative selectivity example. The contact cxb1.1–7×27 is preserved among 9/16 chemokine-GPCR complexes but has low paralog conservation (left). Unfavorable chemokine-GPCR interactions are likely to prevent noncognate chemokine-GPCR pairs. See also Figure S5.

How do individual chemokines and GPCRs achieve selectivity preferences within their respective subfamilies? We hypothesized that chemokines and GPCRs specify selectivity preferences by customizing amino acid identities at key positions in the chemokine-GPCR interface (i.e., “sequence-level changes”) and/or customizing intermolecular residue contacts (i.e., “structure-level changes) (Figure 4A). To investigate this, we performed pairwise comparisons of sequence and structural similarities and differences at the chemokine-GPCR interface for all pairs of human complexes (Figure 4B). Complexes demonstrated a range of overlapping and distinct features, with the most similar complexes sharing 36% of residue contacts (e.g., CCL3-CCR5 and CCL5-CCR5) and 77% sequence identity (e.g., CXCL8-CXCR1 and CXCL8-CXCR2) of interface residues when averaged among paired chemokines and GPCRs (STAR Methods). The most different complexes shared no common residue contacts (e.g., CCL2-CCR2 and CXCL12-ACKR3), and only 15% sequence identity of interface residues (i.e., CCL20-CCR6 and CXCL12-CXCR4).

Next, we grouped each pair of complexes by whether constituent chemokines and GPCRs were (1) part of different subfamilies and had non-overlapping interaction networks (group 1), (2) in the same subfamily but had non-overlapping interaction networks (group 2), or (3) in the same subfamily and had overlapping interaction networks (group 3). Groupings reveal that chemokines or GPCRs sharing interaction networks (group 3) typically employ the highest degree of overlapping sequence and structural features to bind their respective partners. Conversely, chemokines or GPCRs with non-overlapping networks (groups 1 and 2) employ largely non-overlapping sets of sequence and residue contacts (Figure 4B). For instance, CCL3-CCR5 and CCL15-CCR1 complexes (group 3), which have highly overlapping selectivity networks (Figure S1B)—share 32% of contacts and 44% sequence identity at interface positions. Conversely, CCL15-CCR1 and CCL20-CCR6 complexes (group 2)—where neither chemokines (CCL20 and CCL15) nor receptors (CCR5 and CCR6) share any respective binding partners (Figure S1B)—share 15% of contacts and 20% sequence identity at interface positions. This observation suggests that when the chemokine or the receptor share interaction partners, this is mediated through similarity in sequence and structural features (i.e., residue contacts). However, when they do not share interaction partners, it may be driven by changes in sequence and structural features. This pattern among functionally related complexes suggests that selectivity determinants are hierarchically “layered” on top of one another where conserved (generalized), semi-conserved (subfamily-specific), and variable (network-specific) sequence and structural features may determine selective and promiscuous interactions in the chemokine-receptor system (Figure 4C).

We next hypothesized that the observed similarities and differences at each “layer” of selectivity encoding (i.e., generalized, subfamily, and network-specific) might allow chemokines and GPCRs to facilitate binding to some partners (“positive selectivity”) while disfavoring binding to other partners (“negative selectivity”; Figure 4D). To identify instances of positive selectivity, we focused on sequence and structural similarities between CXCL8-CXCR1 and CXCL8-CXCR2 complexes (Figure 4E), which feature the same chemokine bound to different receptors. Among the 61 total residue contacts, 11 (18%) are preserved between complexes and comprise identical chemokine and receptor residues (Figure 4E). This highlights how the conservation of a discrete set of positions among two moderately related receptors—sharing 54% sequence identity at interface positions—can allow them to recognize the same binding partner at a compact, discrete interface (Figure 4F). Analogous results are observed when comparing structures of two moderately related chemokines (i.e., CCL3 and CCL5: 43% sequence identity at interface positions) bound to the same receptor (i.e., CCR5; Figure S5A).

To identify instances of negative selectivity, we identified contacts that are found in a majority of complexes but are comprised chemokine and/or GPCR residues that are poorly conserved among paralogs (Figures S5B and S5C). Among these contacts, cxb1.1(chemokine)-7×27(receptor) displays poor paralog conservation but high ortholog conservation among members of chemokine and receptor families (Figure 4G; Figures S5C and S5D), suggesting that it may help encode chemokine- and GPCR-specific selectivity profiles. Indeed, paired interactions between CXCL12 and noncognate receptor CXCR2 would likely be energetically unfavorable because they would oppose two large, positive side chains in proximity (i.e., CXCL12 Arg12^cxb1.1^ and CXCR2 Arg289^7x27^) (Figure 4G). Likewise, paired interactions between CCL2/CCL3/CCL5 and noncognate receptors such as CCR4 or CCR6 are likely to be energetically unfavorable because they would oppose large, aromatic side chains (chemokine Phe/Tyr^cxb1.1^) with negatively charged Glu279^7x27^ of CCR4 or Glu291^7x27^ of CCR6. Thus, by convoluting multiple types of selectivity information (e.g., positive and negative design) into the chemokine-GPCR interface, chemokines and GPCRs can facilitate interactions with some partners while preventing interactions with others.

### Densely packed selectivity hotspots in unstructured regions and loops

Where are the network-specific selectivity determinants located in chemokine and GPCR structures? Prior analysis (Figures 4E and 4F; Figure S5A) suggests that selectivity determinants are likely to be enriched among contacts that are (1) found in a small set of structures (i.e., structure-level changes) and (2) comprised residues that are poorly conserved among paralogs (i.e., sequence-level changes). We analyzed human chemokine-GPCR complexes and identified contacts that (1) have low structural preservation and (2) are formed by residues that are poorly conserved among paralogs (Figure S5E). Among chemokines, the N terminus demonstrates the highest degree of sequence- and structure-level changes, followed by the b1b2 and cxb1 loops (Figures S5E and S5F). Similarly, among GPCRs, the N terminus demonstrates the region with the highest degree of sequence- and structure-level changes, followed by ECL2 (Figure S5F). In the context of paired interactions among SSEs, most sequence- and structure-level changes involve the chemokine or GPCR N terminus or the GPCR ECL2 (Figure S5G). Consequently, sequence- and structure-level changes are driven by residues in chemokine and receptor N termini and loops, which are thus likely to serve as key regions encoding chemokine- and GPCR-network-specific selectivity preferences.

We next examined whether these regions contribute to chemokine-GPCR recognition using an independent dataset. We mapped saturation mutagenesis data for CXCR4^43^ onto the experimentally guided CXCL12-CXCR4 model^36^ to identify interface regions that most likely encode CXCL12 selectivity. CXCR4 mutations with the largest impact on CXCL12 binding fell into three regions: (1) a three residue stretch in the CXCR4 N terminus (DYD motif; CXCR4 residues 20–22), (2) a set of 11 residues in TMs 1, 2, 3, and 7 and ECL2 that make contacts with a three residue stretch in the CXCL12 N terminus (KPV motif; CXCL12 residues 1–3) (Figure S5H), and (3) conserved receptor residues Cys26^1×22^, Pro27^NTr.Cm1^, and Cys^7x24^ that anchor conserved chemokine-GPCR contacts described in the previous section (Figure 2; Figure 3). In effect, a significant proportion of the CXLC12-CXCR4 interaction is derived from two short residue stretches in chemokine and receptor N termini despite the extensive surface area of the interface. This suggests that a small subset of the chemokine-GPCR interface is responsible for network selectivity, and that selectivity information is enriched in unstructured regions that can be represented as a short linear motif (SLiM) (Figure 4F).

### Identification of SLiMs

**Figure 5.**
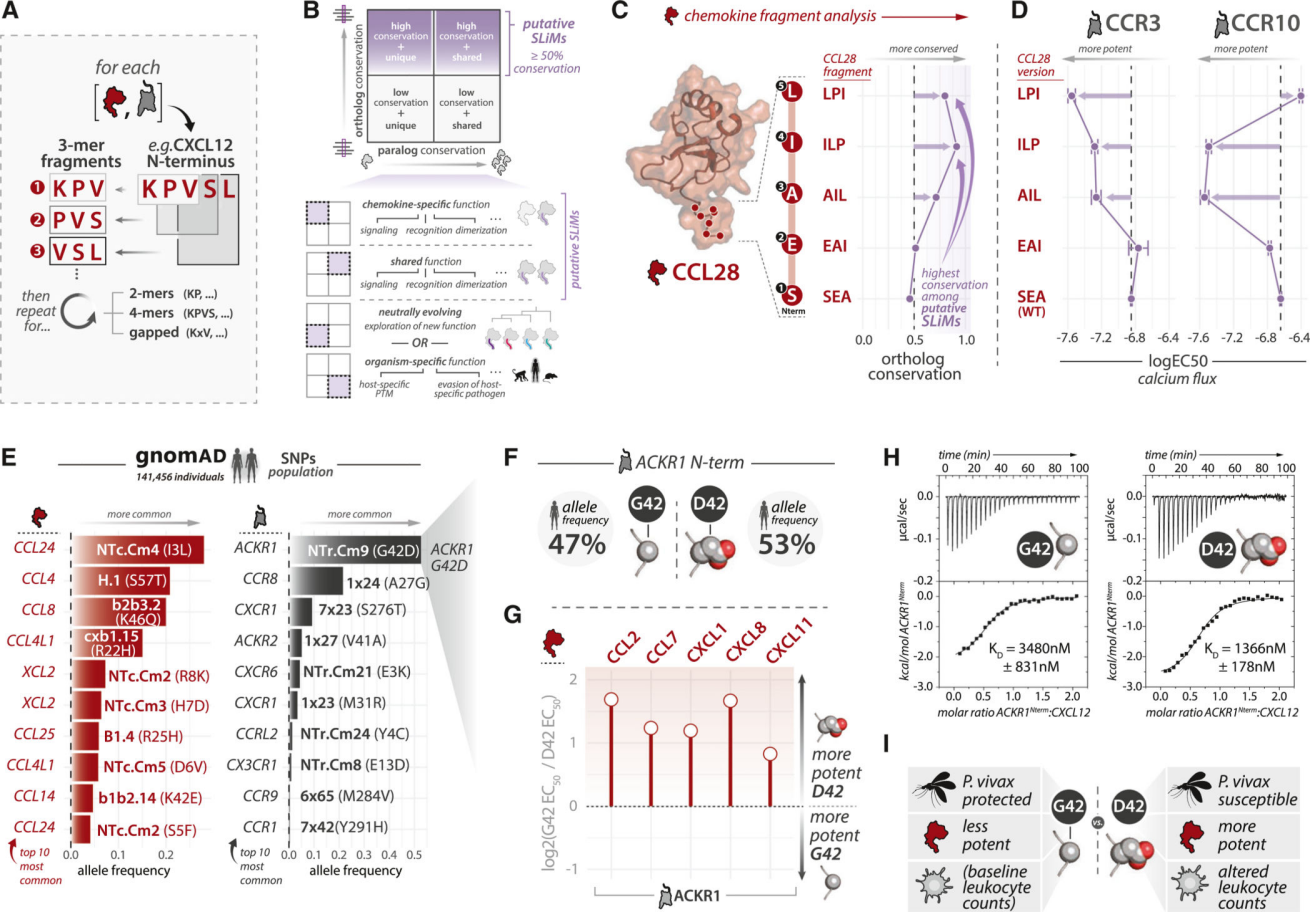
Encoding interactions via SLiMs in unstructured regions (A) Enumeration of all 2-, 3-, and 4-mer fragments for unstructured regions (chemokine/GPCR N termini; GPCR ECL2) using a sliding window approach (STAR Methods). (B) Inferring fragment functional roles based on ortholog/paralog conservation. Fragments conserved in ≥50% of orthologs are called putative SLiMs. (C) CCL28 structure (PDB: 6CWS) depicting N-terminal residues 1–5 and fragment ortholog conservation. (D) LogEC_50_ of CCL28 N-terminal truncation variants values from calcium flux experiments on CCR3 (left) and CCR10 (right) -expressing cells. Error reflects SEM of nonlinear fit of logEC_50_ value. All conditions *n* = 3. See Table S2. (E) Most frequent variants in chemokines (left) and receptors (right) among interface positions from gnomAD. (F) Allele frequency of ACKR1 Gly42 and Asp42. Gly42 allele frequency inferred as 1 Asp42 allele frequency. (G) Log_2_-fold ratio of chemokine EC_50_ values for ACKR1 Asp42 versus Gly42 in bioluminescence resonance energy transfer (BRET)-based binding assay (STAR Methods). See Table S2. (H) ITC performed by injecting 200 μM ACKR1(1–60) Gly42 (left) and Asp42 (right) into WT-CXCL12. Thermograms representative of *n* = 2 replicates. (I) Immune, functional, and phenotypic trade-offs between ACKR1 Gly42 and Asp42 alleles. See also Figures S6 and S7.

Short interaction interfaces in unstructured regions—termed SLiMs—mediate protein-protein interactions (PPIs) in various contexts, including protein complex assembly, subcellular trafficking, and enzymatic recruitment for post-translational modifications.^48^ We postulated that SLiMs in unstructured regions contribute to network-specific interactions. The same SLiM in different positions (within equivalent unstructured regions) of two paralogous proteins can serve the same function, making SLiM identification difficult because their sequences may not be alignable. We developed an *alignment-free* approach to infer short, functional, conserved sequence fragments in chemokine and receptor unstructured N termini and receptor ECL2 that might encode network-specific selectivity preferences (Figure 5A; STAR Methods).

For each sequence in our alignment, we enumerated every 2-mer, 3-mer, 4-mer, and gapped peptide fragment observed in chemokine N termini, receptor N termini, and receptor ECL2 (Figure S6A; STAR Methods). The resulting sequence fragments were then scored for their paralog conservation among other human chemokines and for their ortholog conservation for the same protein across species, agnostic of fragment positioning. Functional relevance of the resulting fragments can be inferred by comparing ortholog and paralog conservation (Figure 5B).^25,49^ For instance, sequence fragments that are conserved among orthologs and shared among multiple paralogs likely confer mutual chemokine recognition of a shared receptor. Fragments that are conserved among orthologs but not among paralogs likely confer a protein-specific function or unique recognition mode of a chemokine or receptor (Figure S6B). Since functionally relevant peptide fragments in either instance are likely to be conserved among chemokine orthologs from different species,^48,50^ we define *putative SLiMs* as peptide fragments with ortholog conservation ≥ 0.5. Relative conservation comparisons at this level help to prioritize fragments with the greatest impact on chemokine or GPCR function. We use the term *fragments* to refer to any 2–4 residue stretch regardless of conservation.

Among putative SLiMs, only 5% (chemokine N terminus), 12% (receptor N terminus), and 1% (receptor ECL2) were shared among five or more chemokines or receptors (Figures S6C and S6D). For instance, the tyrosine sulfation motif “DYG” was shared among 5 receptors N termini, and the CXCR1/2 recognition motif “ELR” was shared among 7 chemokines N termini (Figure S6B). In contrast, a majority of putative SLiMs in chemokine and receptor N termini (60% and 52%, respectively) and receptor ECL2 (70%) were unique to a single chemokine or receptor (Figure S6C).

To investigate how chemokine SLiMs influence receptor recognition, we characterized conservation of peptide fragments in the N terminus of CCL28 and tested the ability of CCL28 variants beginning with various peptide fragment to activate two different receptors: CCR3 and CCR10.^51^ All possible CCL28 N-terminal 3-mer peptides are found exclusively within CCL28 (Figure S6E), consistent with CCL28’s limited and distinct receptor repertoire (Figure S1B). While CCL28 N-terminal peptide fragments have low paralog conservation, they vary in ortholog conservation. The first two 3-mer fragments display an ortholog conservation score of ~0.50 (SEA conservation 0.46; EAI conservation 0.51), and the subsequent three peptide fragments (AIL, ILP, and LPI) have conservation ≥ 0.70, suggesting that the latter fragments may have a larger impact on CCL28 function (Figure 5C). To functionally evaluate CCL28 N-terminal fragments, we tested a series of N-terminal truncation mutants in calcium flux assays in cells expressing receptors CCR3 and CCR10 (Figure 5D; Figure S6F; Table S2). WT CCL28 (SEA; beginning with Ser^NTc.Cm10^) and CCL28-EAI were among the least potent of the tested mutants at both receptors, consistent with the relatively poor conservation of CCL28 3-mer fragments SEA and EAI. In contrast, CCL28-AIL and CCL28-ILP were more potent at both receptors relative to CCL28-SEA and CCL28-EAI. In effect, deletion of Ser^NTc.Cm10^ and Glu^NTc.Cm9^ enhanced CCL28 activation of CCR3 and CCR10, suggesting that these residues, which comprise poorly conserved fragments, negatively impact CCL28 signaling. Notably, while CCL28-LPI showed enhanced activation of CCR3, it showed severely diminished activation of CCR10 (Figure 5D). Together, these data highlight how the same chemokine can differentially modulate activity at different receptors via distinct linear motifs in unstructured regions (Figure S6G).

These analyses support a role for SLiMs in customizing chemokine-GPCR interactions through multiple mechanisms, such as employing the same SLiMs to serve analogous functions (e.g., the ELR motif in CXCR1/2-binding chemokines^34,35^), or unique SLiMs to customize chemokine- or GPCR-specific functions (e.g., distinct CCL28 SLiMs to encode CCR3 and CCR10 recognition). By concentrating chemokine and GPCR selectivity preferences into SLiMs, chemokine and GPCR selectivity can rapidly evolve through emergence or loss of SLiMs rather than re-engineering the entire interface.

### Variant and phenotype mapping to selectivity determinants

We hypothesized that population variants at the chemokine-GPCR interaction interface in the human population might influence selectivity. We gathered variant information for all human chemokines and receptors from three databases: (1) naturally occurring variants from >140,000 healthy individuals from the Genome Aggregation Database (gnomAD)^52^; (2) cancer-associated variants from >10,000 individuals and 33 different cancer types from The Cancer Genome Atlas (TCGA)^53^; and (3) genome-wide statistical associations between variants and disease- or phenotype-associated traits based on data from ~500,000 individuals from the GeneATLAS database (using data from the UK Biobank).^54^ Only missense variants were considered. We mapped variants to CCN and CRN numbering systems and identified genes with the most abundant naturally occurring variants, cancer-associated variants, and phenotypic associations affecting chemokine-GPCR interface residue positions (Figures S6H and S6I; Tables S3, S4, and S5).

**Figure 6.**
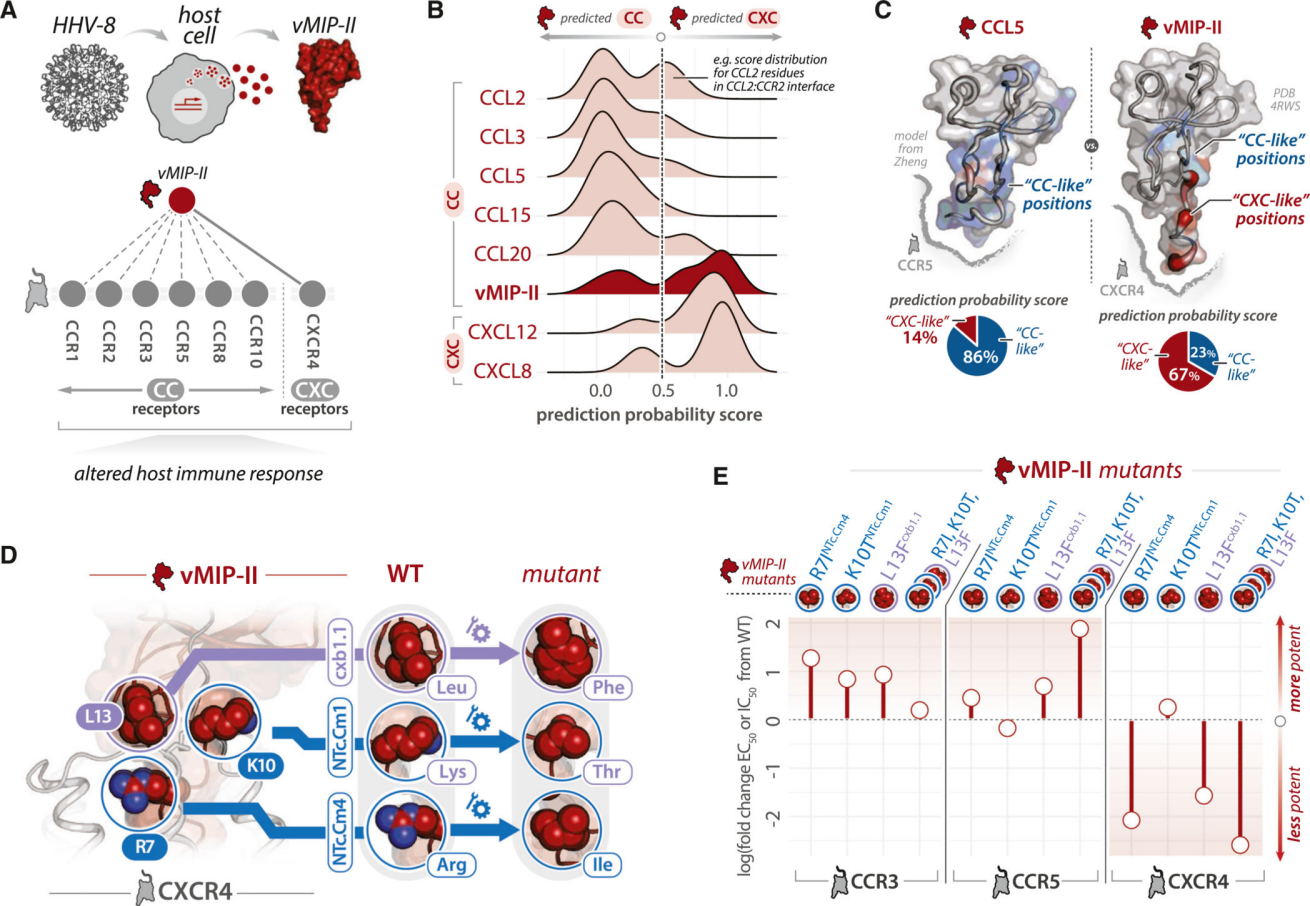
Rational design of altered selectivity using a promiscuous viral chemokine. (A) HHV-8-mediated expression of vMIP-II, which binds CC and CXC receptors to modulate host immunity. (B) Distribution of “prediction probability scores” among chemokine interface residues from chemokine-GPCR complexes by histogram (STAR Methods; Figure S7F). Scores assess likelihood that a queried residue belongs to a CC (i.e., closer to 0) or CXC (i.e., closer to 1) chemokine. Interface residues from Zheng et al.^31^ model were used for CCL5. (C) Percentage interface residues from (B) comprising CC- versus CXC-like residues and mapping onto CCL5/vMIP-II structures. (D) Positions of vMIP-II mutants tested. (E) Log fold change of IC_50_ (or EC_50_ for CCR3) of vMIP-II mutants versus WT for vMIP-II “reversion” mutants, tested at CCR3, CCR5, and CXCR4 in β-arrestin recruitment assays (Figure S7I; STAR Methods). All data *n* = 3. WT vMIP-II and mutants tested as agonists (CCR3) or antagonists (CCR5: in competition with CCL5; CXCR4: in competition with CXCL12). See Table S2. See also Figure S7.

At the gene level, cancer-associated variants were infrequent, with the most variable gene, CCR2, bearing cancer-associated variants in only ~0.3% of tumor samples (Figure S6H; Table S3). In effect, despite established roles for chemokines and receptors in cancer,^55–58^ chemokine-GPCR interface mutations are unlikely to constitute a major oncogenic mechanism. Naturally occurring variants were far more common than cancer-associated variants, with (cumulative) interface variant allele frequencies of ~31% (i.e., CCL24) and ~52% (i.e., ACKR1) for the most variable chemokines and receptors, respectively (Figure 6I; Table S4). Disease- and phenotype-associated variants at interface positions in the UK Biobank dataset revealed 8 chemokines and 6 receptors with statistically significant variant-phenotype associations (Figure S6J; Table S5).^59^ Among these, the chemokine and GPCR with the most associated phenotypes were CCL1 and ACKR1, respectively. Phenotypes most associated with chemokine and receptor variants commonly involve immune-related traits such as blood/immune cell type counts, inflammatory diseases, and infections, consistent with the essential role of chemokines and GPCRs in innate and adaptive immune functions (Table S4).

### Altered selectivity of a common variant in the ACKR1 unstructured N terminus

The most frequently occurring chemokine-GPCR interface variant—ACKR1 Gly42Asp^NTr.Cm9^ (Figure 5E)—influences *Plasmodium vivax* susceptibility.^60,61^ Individuals with the ACKR1 Gly42 ^NTr.Cm9^ allele have resistance to *P. vivax*, whereas individuals with the ACKR1 Asp42^NTr.Cm9^ allele are *P. vivax* susceptible.^62^ ACKR1 is an erythrocyte coreceptor for *P. vivax*, which directly interacts with the sulfated ACKR1 residue Tyr41^NTr.Cm10^ (adjacent to Gly/Asp42 ^NTr.Cm9^) via its Duffy binding protein (PvDBP).^63^ Importantly, sulfation of Tyr41^NTr.Cm10^ is necessary for PvDBP binding to ACKR1.^63,64^ Differences in *P. vivax* susceptibility are thought to have shaped divergent, population-specific allele frequencies. Indeed, while the overall allele frequencies of Gly42^NTr.Cm9^ and Asp42^NTr.Cm9^ are roughly equivalent (Figure 5F), the *P.-vivax*-resistant Gly42^NTr.Cm9^ allele is enriched in Southeast Asia where *P. vivax* endemicity is highest^62^ (Figure S7A).

Since *P. vivax* infection is influenced by adjacent residues in the unstructured ACKR1 N terminus, we hypothesized that Tyr41^NTr.Cm10^ and Gly/Asp42^NTr.Cm9^ function in tandem as a SLiM. We analyzed ortholog and paralog conservation of sequence fragments comprising Tyr41^NTr.Cm10^ and Gly/Asp42 ^NTr.Cm9^ (e.g., fragments YG, YD, DYD, DYG, etc.) in ACKR1 and other chemokine receptors (Figure S7B). YD-containing fragments are shared among 11 receptors versus 6 receptors containing YG fragments, suggesting a broader role for YD-containing fragments. Likewise, YD-containing fragments have higher ortholog conservation among all chemokine receptors and are thus more likely to represent a functional SLiM (Figure S7C).

To evaluate whether Gly42^NTr.Cm9^ versus Asp42 ^NTr.Cm9^ influences ACKR1 chemokine selectivity, we developed a BRET-based ACKR1 binding assay (Figure S7D; STAR Methods). Among CCL2, CCL7, CXCL1, CXCL8, and CXCL11, all showed modest but consistent decreases in potency against ACKR1 Gly42^NTr.Cm9^ as compared with ACKR1 Asp42^NTr.Cm9^ (Figure 5G; Table S2). While CXCL12 binding to ACKR1 was weak in the assays, we assessed CXCL12 binding *in vitro* via isothermal titration calorimetry (ITC) using purified ACKR1 N-terminal peptides (1–60). As with other chemokines, CXCL12 showed modestly reduced potency for ACKR1 Gly42 ^NTr.Cm9^ versus Asp42^NTr.Cm9^ (Figure 5H).

The consistency of observed differences across chemokines suggests that common variants might influence chemokine/GPCR function and phenotype. Indeed, ACKR1 Asp42^NTr.Cm9^ had the most phenotypic associations, and all were related to changes in immune cell counts (Figure S7E). Given key roles for ACKR1 at endothelial surfaces involved in leukocyte trafficking and diapedesis, we propose that binding differences between ACKR1 Gly42^NTr.Cm9^ and Asp42 ^NTr.Cm9^ might modulate circulating lymphocyte counts. More broadly, variations at key selectivity positions within the chemokine-GPCR interface might present functional, phenotypic, evolutionary, and disease-relevant trade-offs (Figure 5I).

### Rewiring selectivity preferences of a promiscuous, viral chemokine

We next tested whether we could leverage the identified selectivity determinants to rationally manipulate chemokine-GPCR selectivity, using the chemokine viral macrophage inflammatory protein II (vMIP-II) as a test case. vMIP-II is secreted by Kaposi’s sarcoma-associated herpesvirus (KSHV, a.k.a. HHV-8)-infected cells to facilitate viral immune evasion.^65,66^ Despite being a CC-subfamily chemokine, vMIP-II acts through receptors in both CC and CXC subfamilies (Figure 6A).

We hypothesized that vMIP-II recognizes receptors of both subfamilies by encoding CC- and CXC-specific sequence features. We evaluated the similarity of vMIP-II residues to those of CC versus CXC subfamily chemokines by using our logistic regression model to assign prediction probability scores (Figure 3C), with human CC and CXC chemokine sequences as controls (STAR Methods). While vMIP-II possesses some CXC-like residues, its sequence predominantly comprises CC-like residues, “true” to its identity as a CC chemokine (Figure S7F; all residues). We next mapped vMIP-II prediction probability scores to the vMIP-II-CXCR4 complex,^39^ which features the viral CC chemokine bound to a receptor of the CXC subfamily. Despite its overall similarity to CC chemokines, vMIP-II preferentially utilizes CXC-like residues to contact CXCR4 (Figures 6B and 6C; interface residues only).

Given the small number of CXC-like residues at the vMIP-II-CXCR4 interface, we hypothesized that targeted mutations of these residues would selectively diminish its interactions with CXCR4 while preserving interactions with CC receptors. We selected vMIP-II Arg7^NTc.Cm4^ and Lys10^NTc.Cm1^, since both residues contact subfamily-specific positions in CXCR4 and are also likely to influence vMIP-II recognition of CC receptors (Figure 3F). Based on similarities of the GPCR interaction profile of vMIP-II to CCL8 (Figure 6A; Table S1), we used CCL8-specific residues Ile7^NTc.Cm4^ and Thr10^NTc.Cm1^ as templates for residue substitutions. Indeed, the proposed mutations are likely to convert them from CXC-like to CC-like residues using logistic regression scoring (Figure S7G). Moreover, the vMIP-II mutations Lys10Thr^NTc.Cm1^ and Arg7Ile^NTc.Cm4^ would introduce putative SLiMs, PxT, and IP, which are found in chemokines with overlapping interaction profiles to vMIP-II (Figure S7H). We introduced Leu13Phe^cxb1.1^, which we predicted would preserve vMIP-II’s interactions with CC receptors but diminish its interactions with CXCR4 by opposing the aromatic Phe^cxb1.1^ with a large, negatively charged Glu277^7x27^ in CXCR4, (i.e., negative selectivity filter; Figure 4G).

All three vMIP-II mutations (Arg7Ile^NTc.Cm4^, Lys10Thr^NTc.Cm1^, and Leu13Phe^cxb1.1^) were tested alone and in tandem alongside WT vMIP-II, in β-arrestin-1 recruitment assays against CCR3, CCR5, and CXCR4 (STAR Methods) (Figures 6D and 6E; Figure S7I; Table S2). Since vMIP-II is a CCR5 and CXCR4 antagonist, vMIP-II WT and variants were tested in concentration-response in the presence of a set concentration of agonist chemokines (i.e., CCL5 and CXCL12, respectively) to observe the potency of vMIP-II at inhibiting β-arrestin recruitment. Since vMIP-II is a CCR3 agonist, we performed standard concentration-response β-arrestin-1 recruitment assays. All three vMIP-II mutants (Arg7Ile^NTc.Cm4^, Lys10Thr^NTc.Cm1^, and Leu13Phe^cxb1.1^), individually or in tandem, showed similar or enhanced potency versus WT vMIP-II at CCR3 and CCR5 (Figure 6D; Figure S7I). The vMIP-II triple mutant, which converts vMIP-II into a mild partial agonist (Figure S7I), showed more drastically enhanced IC_50_ than the individual mutants at CCR5. Jointly, these results suggest how strategic single amino acid substitutions in key, selectivity-determining positions can result in an alteration of function (e.g., enhancement in CCR3 or a gain of function in CCR5).

In contrast to their neutral-to-positive effects at CCR3 and CCR5, vMIP-II mutants caused decreases in potency at CXCR4 in all but one instance. The vMIP-II triple mutant had the largest effect, followed closely by Arg7Ile^NTc.Cm4^ and Leu13Phe^cxb1.1^. These results are consistent with the proposed roles of these mutations in disrupting numerous subtype-specific interactions within CXCR4 (NTc.Cm4) or introducing negative selectivity by opposing a bulky aromatic (cxb1.1) with a large, acidic residue. In contrast, Lys10Thr^NTc.Cm1^ had minimal effects versus WT vMIP-II at CXCR4. Testing vMIP-II mutants in a chemotaxis assay using CXCR4-expressing, human-derived T cells generally mirrored the pharmacology results. All mutants except vMIP-II Lys10Thr^NTc.Cm1^, showed a diminished ability to inhibit chemotaxis relative to WT vMIP-II (Figures S7J–S7L). These results support the notion that the basic principles of chemokine-GPCR selectivity and promiscuity identified here can guide targeted changes to chemokine sequences to modulate binding preferences and effects on cell migration. Moreover, the ability to modulate the selectivity of a viral chemokine for human chemokine receptors using the framework developed here suggests that the hierarchical organization of selectivity determinants is a robust and generalizable concept.

## DISCUSSION

We find that chemokine-GPCR selectivity is hierarchically encoded in conserved, semi-conserved, and poorly conserved sequence and structural elements. Conserved and semi-conserved elements comprise a small fraction of the chemokine-GPCR interface, which is largely dominated by customized interactions involving unstructured regions. The results presented here suggest that different levels of promiscuity are encoded by “tuning” contact similarities among unstructured regions, which contain many 2–4 residue functional hotspots known as SLiMs. In effect, complex patterns of chemokine-GPCR selectivity and promiscuity may emerge from the rapid evolution of unstructured regions.

### Encoding and decoding selectivity using public and private “keys”

**Figure 7.**
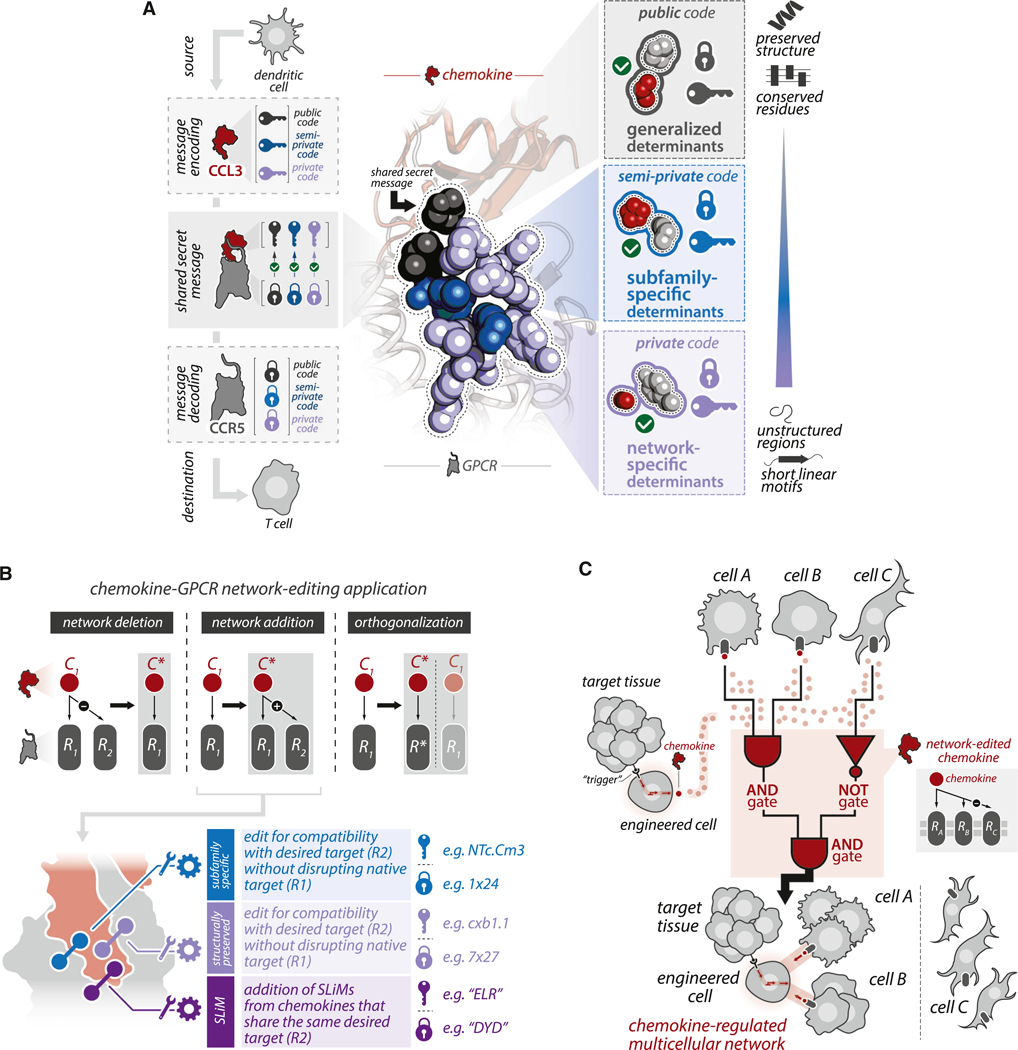
Encoding and decoding chemokine-GPCR selectivity and promiscuity (A) Encryption model for chemokine-GPCR selectivity encoding. (B) Chemokine-GPCR network editing applications. (C) Chemokine regulation of complex multicellular circuits for therapeutic applications.

Selectivity encoding in chemokine-GPCR interactions shares features with digital encryption methods in which two parties (e.g., buyer and seller) can exchange a message (e.g., credit card information) in a public online space using shared public codes and party-specific private codes (Figure 7A). In this scheme, the sender ensures only the intended recipient can unlock the message by providing a composite of public and private codes. The recipient then combines their own public and private codes with those of the sender to form a *shared secret message*, which is distinct from those used by all other possible pairs of parties.

As with digital encryption, the chemokine-GPCR systems distribute selectivity information comprised of generalized (“public”), subfamily-specific (“semi-private”), and network-specific (“private”) sequence and structural elements. During chemokine-GPCR engagement, codes are presented by each party as a composite, where different codes are intermixed among one another on the chemokine (or GPCR) surface and within structured and unstructured regions. In turn, the GPCR binding partner decodes the specific message by structurally positioning its own composites alongside that of chemokine (or vice versa). Importantly, the robustness of encryption results from the distinctiveness of the private codes, which are concentrated in rapidly evolving, unstructured regions. The complexity of private codes (unstructured regions) ensures that every unique pair of chemokines and GPCRs will distinctly encode messages from all other pairs, which may additionally help explain how even closely related chemokines can generate qualitatively distinct signaling profiles.^67^

### Design principles and applications for selectivity-edited chemokines and GPCRs

We leveraged principles of selectivity encoding in the chemokine-GPCR system to design variants of the viral chemokine vMIP-II with a more restricted GPCR selectivity profile. While analogous applications will require context-specific considerations, a generalized framework may help guide design applications. First, one should consider the intended application, including *network edge deletion* (i.e., removing a subset of existing interactions), *network edge addition* (i.e., adding new interactions while preserving existing ones), and *network orthogonalization* (i.e., devising chemokines and GPCRs that exclusively bind one another without engaging native binding partners; Figure 7B). Second, one should gather aligned sequences, including (1) the desired chemokine/GPCR to edit, (2) “in-network” chemokines/GPCRs (e.g., other chemokines that bind the same GPCR as the chemokine of interest), and (3) “out-of-network” chemokines/GPCRs (e.g., chemokines within the same subfamily that do not bind the same GPCR(s) as the chemokine of interest). Third, one should examine residues participating in (1) subfamily-specific interactions (Figure 3F) and (2) structurally preserved contacts between poorly conserved sequence positions (Figure 4A) and identify opportunities to either introduce or remove residues that are likely to participate in unfavorable interactions, using out of network chemokines and GPCRs to guide. Finally, one should identify opportunities to remove or introduce SLiMs, using those contained within the unstructured regions of in-network or out-of-network chemokines/GPCRs to guide (Figure 7B).

Some applications are likely to be more difficult than others. Network edge addition applications may require the introduction of several mutations, some of which may negatively impact the existing function (e.g., native selectivity preferences) and stability of the WT protein.^68^ We also anticipate that network edge addition applications that attempt to introduce interactions to more unrelated chemokines/GPCRs (e.g., CC chemokine interactions with a CXC receptor) or network edge deletion applications that attempt to eliminate interactions among closely related chemokines/GPCRs (e.g., eliminate CCR3 but not CCR5 coupling of CCL5) will be challenging.

How might “network-edited” chemokines and GPCRs be utilized in therapeutic applications? Cell-based therapies, such as engineered chimeric antigen receptor T cells, have a number of limitations, including a lack of precision trafficking, imprecise tumor recognition, and transient efficacy (e.g., T cell “exhaustion”).^69^ Incorporating network-edited chemokines and GPCRs into the design of cell-based therapies could help overcome these limitations, for instance, by enhancing targeting prior to activation.^70,71^ As master regulators of cell migration, chemokines and GPCRs have the potential to function as programmable logic gates that control cell migration in complex multicellular circuits (Figure 7C).^72^

### Limitations of the study

Chemokine-GPCR selectivity is modulated by tissue-specific expression patterns,^73–75^ chemokine-glycosaminoglycan (GAG) interactions,^76^ oligomerization,^77^ and other factors not considered here. Future work should investigate selectivity encoding among ACKRs, which may have distinct chemokine-binding modes.^37,78^ Structures of human chemokine-GPCR complexes that were published during the late stages of manuscript preparation or review^78–86^ could not be included in our analysis. Some structures used in this study have limited resolution of interface interactions involving unstructured regions, which play important roles in recognition.^24^ Regarding SLiMs, we employ conservation as a proxy for functional importance; however, organism-specific motifs can emerge, especially when unique selective pressures such as host-pathogen interactions are considered.^87,88^ Relatedly, the specific functional roles of SLiMs (e.g., contributions to specific binding partner interactions) cannot be distinguished from other functions by our method alone due to interface customization among complexes and contributions of conformational dynamics to binding.^89,90^ Additional experiments will be needed to explore applicability of selectivity principles to chemokine-GPCR interactions not tested here. For instance, future efforts that pursue network edge addition applications, applications using human-only chemokine-GPCR networks, and network orthogonalization, among others, can further validate the findings and the presented framework.

## RESOURCE AVAILABILITY

### Lead contact

Further information and requests for resources and reagents should be directed to and will be fulfilled by the lead contact, M. Madan Babu (madan.babu@stjude.org).

### Materials availability

Further information and requests for resources and reagents should be directed to and will be fulfilled by the lead contact.

### Data and code availability

All data reported in this paper will be shared by the lead contact upon request. All original code has been deposited at github.com and is publicly available at https://github.com/andrewbkleist/chemokine_gpcr_encoding as of the date of publication. Any additional information required to re-analyze the data reported in this paper is available from the lead contact upon request.

## STAR★METHODS

### EXPERIMENTAL MODEL AND STUDY PARTICIPANT DETAILS

#### Cell lines

Cell lines used in this study include HEK293T cells (ACKR1 binding assays; CCR5 and CXCR4 β-arrestin recruitment assays; Abcam, ab255449), Chem-1 Ready-to-Assay^™^ CCR3 Chemokine Receptor Frozen Cells (CCR3 calcium flux assays; Eurofins, HTS008RTA), Chem-1 Ready-to-Assay^™^ CCR10 Chemokine Receptor Frozen Cells CCR10 calcium flux assays; Eurofins, HTS014RTA), and healthy donor T cells (derived from human peripheral blood mononuclear cells, PBMCs). HEK293T cells were grown in Dulbecco’s Modified Eagle Medium (DMEM)/10% fetal bovine serum at 37°C with 5% CO2. HEK293T cells were checked for mycoplasma contamination upon receipt (Venor^®^GeM OneStep kit; Cat. No.: 11–8100, Minerva Biolabs GmbH) and routinely every 3 months. Ready-to-Assay^™^ CCR3 and CCR10 Chemokine Receptor Frozen Cells were prepared per manufacturer instructions by thawing supplied frozen cell stocks, washing, resuspending using supplied reagents, and plating in 96-well plate for 24h for calcium flux assays (see below for assay details). CCR3/CCR10 cells were used shortly after receipt and were not specifically tested for mycoplasma. T cells were isolated from PBMCs by direct enrichment of leukapheresis product by immunomagnetic separation using CD4+/CD8+ microbeads (Miltenyi, Germany), an LS column (Miltenyi), and an AutoMACS separator (Miltenyi). Cells were then frozen in Recovery^™^ Cell Culture freezing media (Gibco, NY, USA) at 1 × 10^7^ cells/mL in a 1 ml cryovial. Frozen stocks of PBMC-enriched T cells were grown in RPMI 1640 (Cytiva, UT, USA) supplemented with 10% FBS (GE Healthcare, Chicago, USA), 1% GlutaMAX (Thermo Fisher Scientific, MA, USA), and cytokines IL7 and IL15 (10 ng/mL each) (Biological Resources Branch, National Cancer Institute, Frederick, MD, USA, and PeproTech, Rocky Hill, NJ, USA) prior to experimental setup. PBMCs were obtained from deidentified healthy donor pheresis products and are exempt from IRB permissions. Enriched T cells were authenticated by measuring CD4, CD8, and CXCR4 expression (among other markers, see below) by flow cytometry. All other cell types were purchased directly from suppliers prior to experimental use and were thus not independently authenticated.

### METHOD DETAILS

#### Chemokine-GPCR interaction network

An initial matrix of reported chemokine-GPCR interactions was established using published interaction matrices from review articles^108–113^ and pairwise chemokine-GPCR interactions extracted from CellphoneDB.^74,114^ Additional interactions and annotations were incorporated during table annotation. Each entry (i.e. row) encompasses a single chemokine-GPCR interaction associated with a specific reference, such that two different references reporting the same interaction are listed as two entries. For each entry in which ligand binding data, signaling data (e.g. calcium flux or cAMP accumulation), or chemotaxis data were presented, an “Interaction Strength” was assigned, with “3” assigned for ligand binding Kd or Ki, ligand binding EC50 or IC50, signaling data EC50 or IC50, and/or maximal chemotaxis values ≤ 100nM; “2” assigned for the same parameters with values > 100nM and ≤ 1000nM; “1” assigned for the same parameters with values > 1000nM; and “0” assigned if the interaction was tested but no effect was observed. Each entry was also assigned an “Evidence Grade”, with “A” assigned for quantitative evidence derived from dose-response testing (e.g. binding Kd, binding or signaling EC50, IC50); “B” assigned for semi-quantitative evidence derived from testing of numerous ligand concentration points but without a derivation of a quantitative summary statistic such as EC50/IC50, etc. (e.g., dose of maximal chemotaxis among three tested doses); “C” assigned for qualitative evidence (e.g., lack of response noted in raw calcium flux traces from stimulation using a single concentration); and “D” assigned for indirect evidence (e.g., ligand-stimulated chemotaxis of native cells known to express receptor of interest). Claims made based on evidence not provided (e.g., “data not shown”) were not considered. For interactions supported by evidence in multiple papers, not all instances were compiled. Data from transfected cells were prioritized over that from primary cells due to higher confidence of receptor expression profiles in the former.

Interactions with an Interaction Strength ≥ 2 and supported by at least one reference with Evidence Grade C were considered for analyses that incorporate network information (Figure 1D; Figure S1B; Figure 3B). Network information is available as Table S1. The network representation of CC and CXC chemokine receptors (Figure 3B) incorporates human CC and CXC chemokines and GPCRs from Table S1 (inclusive of interactions with an Interaction Strength ≥ 2 and supported by at least one reference with Evidence Grade C). The chemokine-GPCR network representations were generated with Cytoscape.

#### Endogenous ligand and receptor numbers among GPCR families from Classes A, B, C, and F

Endogenous ligand and receptor data were downloaded from the International Union of Basic and Clinical Pharmacology/British Pharmacological Society (IUPHAR/BPS) Guide to Pharmacology (http://www.guidetopharmacology.org/GRAC/ReceptorFamiliesForward?type=GPCR) from the GPCR list page.^115^ Data for Class A, Class B, Class C, Class Frizzled, and Adhesion class, and ligand sets were manually edited to retain only human ligands. All data were processed and plotted in R.

#### Chemokine sequence acquisition and sequence alignment

##### Acquisition of human chemokine paralog sequences and alignment

Human chemokine paralogs were compiled from Pfam^116^ and Uniprot^117^ yielding the following list of 46 human chemokines: CCL1, CCL2, CCL3L1, CCL3, CCL4L1, CCL4, CCL5, CCL7, CCL8, CCL11, CCL13, CCL14, CCL15, CCL16, CCL17, CCL18, CCL19, CCL20, CCL21, CCL22, CCL23, CCL24, CCL25, CCL26, CCL27, CCL28, CXCL1, CXCL2, CXCL3, CXCL4L1, CXCL4, CXCL5, CXCL6, CXCL7, CXCL8, CXCL9, CXCL10, CXCL11, CXCL12, CXCL13, CXCL14, CXCL16, CXCL17, CX3CL1, XCL1, XCL2. The chemokines CCL3L3 and CCL4L2 were found in Ensembl but not Uniprot and were thus excluded from analysis. For each of the 46 human chemokine paralogs, full length, unprocessed sequences were downloaded from www.uniprot.org by selecting the listed ‘canonical’ sequence.

Sequence alignment of human chemokine paralogs proceeded as follows. First, experimental structures were downloaded from the PDB for the 35 of 46 human chemokines having at least one structure at the time of initial alignment construction (March 2017), which included the following PDBs (PDB ID is listed with the selected chain indicated after the underscore): 1EL0_A, 1DOK_A, 3FPU_B, 1JE4_A, 1U4P_A, 1NCV_A, 1ESR_A, 1EOT_A, 2RA4_A, 2Q8R_A, 2HCC_A, 1NR4_A, 4MHE_A, 2MP1_A, 1M8A_A, 5EKI_A, 1G91_A, 1EIG_A, 1G2S_A, 2KUM_A, 1MSG_A, 1QNK_A, 1RHP_A, 2MGS_A, 1NAP_A, 5D14_A, 1LV9_A, 1RJT_A, 2J7Z_A, 4ZAI_A, 2HDL_A, 4XT1_B, 1J9O_A, 4HSV_A, 6CWS_A. Waters, cofactors, and other non-protein components were removed using the ‘trim’ command from the “Bio3D” package in R.^98^ Second, trimmed PDBs were used to generate a structure-based sequence alignment via MUSTANG.^105^ Third, for the 25 sequences for which structures were available, Uniprot sequences substituted into the structure-based alignment for the corresponding chemokine while preserving the overall alignment. Fourth, full length Uniprot sequences for all 46 chemokine paralogs were independently aligned via MUSCLE.^104^ Fifth, sequences for each of the 10 chemokines lacking structures were paired with those of closely related chemokines for which structures were available from the MUSCLE alignment. The two sequences were then adjusted in tandem to align the sequence-based MUSCLE alignment to the structure-based MUSTANG alignment using sequence represented in both MUSTANG and MUSCLE alignments as a “bridge”. Sixth, the alignment containing all 46 human chemokine sequences was manually inspected and refined. Sequence visualization and manual refinement was performed in Jalview.^101^

Since chemokine N- and C-termini are unstructured, they were not considered during structure (MUSTANG)- and sequence (MUSCLE)-based alignment steps. Instead, they were positioned without gaps adjacent to the first chemokine cysteine that is involved in disulfide bonding (N-terminus) and the C-terminal helix (C-terminus), respectively. The boundaries of the N-terminal, core, and C-terminal master alignments were chosen by demarcating the core as spanning the first Cys of the Cys motif (i.e., CC, CXC, CX3C, XC) to the last residue of the helix as defined by CXCL12 residue Ala65 (numbered from the CXCL12 N-terminus starting with 1-KPVS-…), which is defined as the end of the helix in the CXCL12 using PDB ID 2J7Z. Residues on either side of these boundaries were defined as belonging to the N- and C-termini, respectively. While the N- and C-terminal regions are unstructured, inspection of isolated chemokine-GPCR complex structures indicates that the core-adjacent residues within these regions are constrained by proximity to the structured core, such that alignment positions of core-adjacent residues are likely to encode functionally relevant relationships between chemokines.

##### Acquisition and alignment of chemokine ortholog sequences

For each of the 46 human chemokine paralogs, 1:1 orthologs were acquired by searching the Orthologous MAtrix (OMA) database using human canonical Uniprot sequences.^93,118^ For the chemokines CCL14, CCL15, CCL19, CCL23, CXCL12, CXCL16, and CX3CL1, the given human sequence from OMA did not match the human canonical sequence from Uniprot, so the OMA sequence was used. OMA did not report 1:1 orthologs for the chemokines CCL4, CCL4L1, CCL5, XCL1, and XCL2. 1:1 orthologs for CCL4 and CCL5 (but not CCL4L1, XCL1, and XCL2) were identified via Ensembl Compara.^119^ The final alignment contained orthologs lists for 43/46 human paralogs with 3 or more orthologs per chemokine. Sequences were downloaded from OMA between April 21, 2017 and June 24, 2017 and from Ensembl Compara on June 24, 2017. To create a master alignment of all ortholog and paralog sequences, ortholog sequences for each chemokine were independently aligned using MUSCLE, then mapped to the structure-based alignment of human chemokine paralogs using the human paralog sequence in both alignments as a “bridge”. The master alignment of chemokine sequences contains 1058 sequences, comprised of 46 human chemokine paralogs and ortholog sets for 43/46 human paralogs. After making the master alignment, additional alignments were generated by selecting different subsets of chemokine sequences for conservation analysis. The following alignments were utilized: (1) a *master alignment* containing 1058 sequences, including all 46 human paralog sequences and sets of ortholog sequences for 43/46 chemokines; (2) a *human paralog alignment* containing 46 human chemokine paralog sequences; (3) *subfamily-specific human paralog alignments* containing 26 (CC subfamily) or 17 (CXC subfamily) human chemokine sequences, (4) a *CC-/CXC-alignment* containing only CC and CXC ortholog and paralog sequences (i.e., excluding CX3CL1, XCL1, and XCL2 orthologs/paralogs); and (5) *chemokine-specific ortholog alignments* containing ortholog sequences for each of the 43/46 human chemokines for which at least 3 one-to-one orthologs could be obtained.

##### Alignment of N-termini for chemokine ortholog sequences

For the 43/46 chemokines having 1:1 orthologs, ortholog N-terminal sequences (i.e., up to but not including the first conserved cysteine) were aligned using the KMAD knowledge-based multiple sequence alignment algorithm, which is optimized for enrichment of short linear motifs (SLiMs) in unstructured regions.^102^ The KMAD algorithm produces “insertion free” alignments by removing residues that fail to match motifs identified in a reference sequence. Each set of ortholog sequences were aligned independently, using the human sequence as the reference sequence.

#### Common chemokine numbering scheme

Common chemokine numbering (CCN) positions (Figure S2) are defined as follows: each secondary structural element (SSE) is given an identifier (i.e., NTc = N-terminus; CX = Cys region; cxb1 = N-loop; B1 = β1-strand; b2b2 = 30s-loop; B2 = β2-strand; b2b3 = 40s-loop; B3 = β3-strand; b3h = 50s-loop; H = helix; CT = C-terminus) and each position within the SSE is given an index (e.g., b1b2.3 = third residue in the 30s-loop), analogous to previous studies on G proteins, GPCRs, arrestin, and TATA-box-binding protein (TBP).^26,120–122^ Loops are designated using the two adjacent structured SSEs in lowercase lettering (e.g., the loop region referred to as the “30s-loop” occurs between the β2- and β3-strands and is designated as b2b3). N-terminal positions are named using the SSE identifier NTc (i.e. N-terminus of chemokine), followed by a period and a modified numerical index prefaced by “Cm”, to indicate that the residue position in question is at position “cysteine minus” the indicated number of residues. For instance, “NTc.Cm3” indicates a residue in the chemokine N-terminus three residues preceding the first disulfide-bonding cysteine.

#### Chemokine receptor sequence acquisition, sequence alignment, and common numbering

Compilation of human chemokine receptor paralogs from Pfam,^116^ Uniprot,^117^ and GPCRdb^26^ yielded the following list of human chemokine receptors: CCR1, CCR2, CCR3, CCR4, CCR5, CCR6, CCR7, CCR8, CCR9, CCR10, CXCR1, CXCR2, CXCR3, CXCR4, CXCR5, CXCR6, CX3C1, XCR1, ACKR1, ACKR2, ACKR3, ACKR4, CCRL2. At least one study reports a seventh CXC family receptor (GPR35, referred to as “CXCR8”),^123^ however this was disputed in another report^124^ and thus excluded. Human chemokine receptor paralog sequences were downloaded from Uniprot. For each of the 23 human chemokine receptor paralogs, 1:1 ortholog sequences were downloaded from OMA.^93,118^ In cases for which OMA and Uniprot canonical sequences did not match, the OMA human sequence was used. Ortholog sequences were independently aligned using MUSCLE, resulting in 23 sequence alignments (i.e., one alignment containing multiple chemokine receptor orthologs for each of the 23 human chemokine receptor paralogs). Insertions in chemokine receptor ortholog sequences that were not present in the human sequence were remove since this study focuses on the human chemokine-GPCR system. The 23 ortholog sequence alignments were manually maped to the structure-based alignment of the chemokine receptor family downloaded from GPCRdb^26^ by using human chemokine receptor sequences as a “bridge”. The resulting chemokine receptor master alignment contains 951 sequences, comprised of 23 human chemokine receptor paralog sequences, and ortholog sequences for 23/23 human paralogs. Using the master alignment, additional alignments were generated by selecting different subsets of sequences for analysis. The following alignments were utilized: (1) a *master alignment* containing 951 sequences, including all 23 human paralog sequences and sets of ortholog sequences for all 23 human receptors; (2) a *human paralog alignment* containing only the 23 human receptor paralog sequences; (3) *subfamily-specific human paralog alignments* containing 10 (CC family) or 6 (CXC family) human receptor sequences; (4) a *CC-/CXC- subset of the master alignment* contains only CC and CXC orthologs and paralogs (i.e., excluding ACKR1–4, CCRL2, XCR1, and CX3CR1); and (5) *receptor-specific ortholog alignments* containing ortholog sequences for each of the 23 human receptors.

#### Common chemokine receptor numbering scheme

The existing GPCRdb numbering was used to refer to structurally equivalent positions across GPCR structures with minor modifications. The GPCRdb alignment used at the time of sequence acquisition designates the first common numbering position as occurring in transmembrane domain 1 (TM1) at position 24 (GPCRdb numbering: 1×24). We extended the common numbering scheme toward the N-terminus of our master chemokine receptor alignment to include a conserved N-terminal cysteine. The conserved N-terminal cysteine forms a disulfide bond with a cysteine in TM7 in chemokine receptors (common chemokine receptor numbering [CRN] position 7×24).^27,44,125^ To implement this modification, conserved cysteines were manually aligned in the master alignment (i.e. the alignment containing all chemokine receptor paralogs and orthologs) and the N-terminal cysteine residue was designated 1×22. The residues following 1×22 were then aligned without gaps adjacent to the cysteine and designated as position 1×23. While existing GPCRdb assignments were not changed, alignments for which gaps were present in the existing GPCRdb alignment were modified in our CRN to move unassigned residues into gapped TM1 positions. For example, in the GPCRdb alignment for CCR2, Lys34, Phe35, and Asp36 are unassigned (and positioned to the N-terminus of the start of TM1) and CCR2 has gaps at positions 1×24–1×26. In our CRN, we moved these three residues into the gapped region, yielding Lys34^1×24^, Phe35^1×25^, and Asp36^1×26^ CRN assignments. As with the chemokine sequence alignment, N-terminal positions were “stacked” against Cys^1×22^ and designated with the SSE identifier NTr (i.e. N-terminus of receptor) followed by a period and a modified index prefaced by “Cm” to indicate that the residue position in question is at position “cysteine minus” the indicated number of residues. For instance, “NTr.Cm3” refers to the residue in the receptor N-terminus that precedes the first disulfide-bonding cysteine by three residues. The chemokine receptor alignment in the region of extracellular loop 2 (ECL2) was left unmodified relative to the existing GPCRdb alignment between positions 4×56–45×52. Residue positions succeeding 4×56 and preceding 45×50 were assigned identifiers ECL2.1-ECL2.13. The GPCRdb alignment in this region adjusts sequence positions without gaps and coming after TM4, such that positions after 4×65 and prior to 45×50 are non-structurally equivalent. As such, conservation scoring of this region was not performed. The region between the residue position succeeding 45×52 and the residue preceding 5×31 were modified by removing all gaps and thus “collapsing” all residues coming after the GPCRdb-aligned positions 45×50–45×52 toward the C-terminal end of this region. Following our convention used for chemokine receptor N-terminus, residues succeeding 45×52 were designated with SSE identifier ECL2 followed by a period and a modified index beginning with “Cp” to indicated “cysteine plus.” Since the conserved ECL2 Cys occurs at position 45×50 and GPCRdb provides alignments through 45×52, the modified nomenclature starts at ECL2.Cp3 in our CRN. At ECL3, CCR2 was adjusted to move Cys277 into position 7×24, which contains the conserved Cys that is disulfide-bonded to Cys^1×22^ in all other chemokine receptors except CXCR6 – the only chemokine receptor that does not have a Cys at this position or at the N-terminus-TM1 junction.

#### Conservation scoring of chemokine and GPCR orthologs and paralogs

CCN- and CRN-specific conservation scores were generated for the following sets of sequences for both chemokines and chemokine GPCRs: human chemokine and GPCR paralogs (1 alignment each), human CC paralogs (1 alignment each), human CXC paralogs (1 alignment each), human CC and CXC paralogs combined (1 alignment each), human atypical chemokine receptor (ACKR) paralog sequences only (1 GPCR alignment), and chemokine and GPCR orthologs (43 and 23 alignments, respectively). Conservation was calculated on a scale from 0 to 1 using the *trident* scoring algorithm from the software MstatX using default settings (https://github.com/gcollet/MstatX), with 0 representing no conservation and 1 representing complete identity.^103^

#### Subfamily-specific classification of chemokine and GPCR residue positions

The predictive accuracy of chemokine (and GPCR) alignment positions for discriminating whether a chemokine (or GPCR) sequence belongs to CC versus CXC subfamilies was evaluated using a logistic regression model. First, sequences in each master alignment (i.e., all aligned sequences including all human paralogs and respective 1:1 orthologs) were labelled according to chemokine (or GPCR) subfamily, and non-CC- and CXC-subfamily sequences were removed. Second, logistic regression models were trained using 80% of CC and CXC sequences, focusing on a single residue position at a time as input features. Logistic regression was performed using the *glm* function in base R. Since CC sequences are more abundant than CXC sequences in both chemokine and GPCR master alignments, CC sequences were randomly down sampled to create equal training set classes. Each position-specific, trained model was evaluated for its accuracy at predicting the correct chemokine family (i.e., CC vs. CXC) of the remaining 20% of sequences with blinded subfamily labels. For chemokines, of the 1058 total sequences in the master alignment, 1018 sequences were of CC or CXC chemokines, which was down sampled to produce 421 CC and CXC sequences each (842 sequences total). 672 of these 842 chemokine sequences were used for in the training set, and 170 sequences were used in the test set. For receptors, of the 951 total sequences in the master alignment, 647 were of CC or CXC chemokines, which was down sampled to produce 226 CC and CXC sequences each (452 sequences total). 360 of these 452 receptor sequences were used in the training set, and 92 sequences were used in the test set. Training and test set sequences were randomly partitioned from total available sequences in triplicate by using a random seed generator in base R. For each position, a “subfamily score” was generated by taking the mean predictive accuracy of the logistic regression model at accurately predicting the subfamily (i.e. CC versus CXC) of a given, unlabelled test set sequence at the position in question across three replicates. Subfamily-predictive positions were defined as positions with subfamily scores ≥75% (represented in Figure S3B). Logistic regression scoring of unknown, test set sequences required that the model was trained on sequences containing any residues that might appear in a test set sequence at a particular position. To ensure the model was trained on sequences with comprehensive sampling of residues, random seeds that partition training and test set sequences were empirically adjusted such that training sequences were inclusive of all residues that would appear in the test set. Adjustments were done blinded from classification accuracy.

#### Selection of chemokine-GPCR complexes

Chemokine-GPCR complex structures were downloaded from www.rcsb.org for the following complexes for structural analysis: CCL2-CCR2 (7XA3),^29^ CCL3-CCR5 (7F1T),^30^ CCL5[5P7]-CCR5 (5UIW),^31^ CCL5[6P4]-CCR5 (7O7F),^32^ CCL5-CCR5 (7F1R),^30^ CCL15-CCR1 (7VL9),^28^ CCL20-CCR6 (6WWZ),^33^ CXCL8-CXCR1 (8IC0),^34^ CXCL8-CXCR2 (6LFO),^35^ CXCL12-ACKR3 (7SK3),^37^ CX3CL1-CX3CR1 (7XBX),^38^ vMIP-II-CXCR4 (4RWS),^39^ CX3CL1-US28 (4XT1),^40^ and CX3CL1.35-US28 (5WB2).^41^

In instances in which multiple closely related structures were available, a single structure was chosen as representative of that chemokine-GPCR interaction. For instance, gross inspection of structurally superposed complexes of CCL15 (26–92)-CCR1 (7VL9) and CCL15 (27–92)-CCR1 (7VLA) reveals almost identical positioning of CCL15 and CCR1, such that the latter complex is likely to recapitulate the same residue-residue interactions as the former apart from those formed by the first CCL15 residue which is truncated in the second complex. As such, only the former complex was included. Similarly, inspection of monomeric (6LFO)- and dimeric (6LFM) CXCL8-CXCR2 complexes reveals almost identical positioning of CXCL8 and CXCR2 except for the second CXCL8 protomer, which is largely displaced from the CXCR2 interface. Given the similarity of binding poses, only the monomeric CXCL8-CXCR2 complex was included. While multiple versions of the CCL5-CCR5 complex are included in our analyses, the CCL5 ligand variants in each case have distinct N-terminal sequences from one another and have unique functional properties,^31,32^ so we considered these to be non-redundant in some instances. Of note, the WT CCL5-CCR5 (7F1R) complex has low resolution and was unable to be modeled at key regions of the chemokine-GPCR interface including the CCR5 N-terminus and N-terminal portion of TM1 and part of the CCL5 “N-loop” (i.e., CCN: cxb1).

In addition to these structures, published full length models of CCL5-CCR5 (model PDB file available as “Document S2”: https://www.cell.com/immunity/fulltext/S1074–7613(17)30218–2?_returnURL=https%3A%2F%2Flinkinghub.elsevier.com%2Fretrieve%2Fpii%2FS1074761317302182%3Fshowall%3Dtrue#supplementaryMaterial)^31^ and CXCL12-CXCR4 (model PDB file available as “S2 Data”: https://journals.plos.org/plosbiology/article?id=10.1371/journal.pbio.3000656#sec020)^36^ complexes were included in analyses. Both models are based on experimental data (CCL5-CCR5: based on CCL5[5P7] experimental structure; CXCL12-CXCR4: based on disulfide cross-linking studies to derive modeling restraints) and were included to expand the number of chemokine-GPCR complexes.

The CXCL8-CXCR1 complex structure (8IC0)^34^ was published after completion of initial data analysis and experimental testing of CC- versus CXC-subfamily determinants (Figure 3H). This complex was incorporated into analyses presented in Figures 1, 2, and 3 and associated Supplemental Figures. The inclusion of this complex did not alter conclusions of any analyses performed in its absence other than minor changes to total number of contacts and unique subfamily contacts presented in Figure 3F.

#### Residue contact calculations and contact fingerprinting

To identify complex-specific residue-residue interactions, waters, cofactors, other non-protein components, and hydrogens (in the case of CCL5-CCR5 and CXCL12-CXCR4 models as well as soluble chemokine-GPCR complexes) were removed from PDBs using the ‘trim’ command from the “Bio3D” package in R.^98^ Intermolecular residue-residue contacts were calculated for each complex using Protein Contact Atlas (https://www.mrc-lmb.cam.ac.uk/pca/index.html) with default settings.^42^ Custom scripts were written in R to assign CCN and CRN designations for chemokines and GPCRs to residues involved in intermolecular contacts. Among the 16 analyzed complexes, 953 contacts were identified, among which 442 occurred between distinct CCN and CRN positions. Residue-residue contacts (referred to simply as “contacts”) were depicted on chemokine-GPCR complex structures by appending chemokine-GPCR complex PDB files with CONECT records specifying intermolecular contacts between chemokine and GPCR Cα atoms. Custom scripts were written in R incorporating “Bio3D” functions to generate complex-specific CONECT records. PyMol was used to represent contacts.

The functional role of residue-residue contacts can be evaluated by assessing the extent to which a contact between structurally equivalent ligand and receptor residues are preserved among different structural complexes. For each residue-residue contact (listed in terms of CCN and CRN), the presence or absence of that contact among all 16 chemokine-GPCR complexes was recorded. The set of all residue-residue contacts for one or more complexes is referred to as a “contact fingerprint”.^126^ We make a distinction between the terms *preservation* and *conservation*: with *preservation* used to designate residue-residue interactions found in numerous complexes (e.g., “structural preservation”), and *conservation* used to designate the utilization of residues with sequence identity or similarity among different chemokines or receptors at a given alignment (i.e. CCN or CRN) position (e.g., “sequence conservation”).

#### Structural variability of chemokine-GPCR complexes

Structural variability among chemokine-GPCR complexes was evaluated as follows. First structurally equivalent CCN and CRN positions were assigned to chemokines and receptors comprising each complex using associated PDB files and master sequence alignments. Second, for each pair of complexes, chemokines (or GPCRs) were structurally superposed using Cα atoms for CCN (or CRN) positions shared in both molecules. Structural superposition was performed using the “fix.xyz” function from the “Bio3D” package in R.^98^ Third, root-mean-square deviation (RMSD) was calculated for each pair of structurally equivalent Cα atoms (i.e., equivalent CCN or CRN designtaions) using the Bio3D “rmsd” function. Mean pairwise RMSD for each pair of molecules was calculated by taking the average of all pairwise Cα RMSD values. Pairwise RMSD of full chemokine-GPCR complexes was performed analogously to calculation of pairwise RMSD for chemokines or GPCRs alone, except chemokine-GPCR complexes were first structurally superposed using receptor coordinates (using Cα atoms belonging to shared CRN positions of the receptor within each complex), followed by calculation of pairwise RMSD for Cα atoms belonging to shared CCN positions of chemokines.

#### Shared sequence and structural features of chemokine-GPCR complexes

Intermolecular, chemokine-GPCR residue-residue contacts from all 16 complexes were annotated with position-specific sequence features for chemokine and GPCR positions involved in the contact, including: paralog conservation, CC paralog conservation, CXC paralog conservation, CC and CXC paralog conservation, ACKR paralog conservation (GPCRs only), non-ACKR paralog conservation (GPCRs only), and ortholog conservation (for the specific chemokine and GPCR involved; only annotated for human chemokines/GPCRs and at residue positions that were unmodified, e.g. excluding N-terminal residues in CCL5[5P7] and CCL5 [6P4]). Chemokine and GPCR positions involved in contacts were also annotated with subfamily scores and subfamily score standard deviations.

Chemokine-GPCR contacts occurring between sequence conserved positions were identified by filtering the list of all 953 contacts for those contacts occurring between chemokine and GPCR positions with paralog conservation scores ≥ 0.50. The uniqueness of chemokine orientation in the CXCL12-ACKR3 complex relative to that observed on all other complexes^37^ suggests that ACKRs may couple chemokines in structurally distinct ways, thereby employing structurally analogous residue positions for distinct chemokine interactions relative to non-ACKR (i.e. “conventional”) counterparts. As such, we used paralog conservation scores for conventional (i.e. non-ACKR) non-ACKR human chemokine receptor paralogs to identify pairwise contacts among conserved chemokine and GPCR residue positions. Among the 442 chemokine-GPCR contacts occurring between distinct CCN and CRN positions, 5 were identified between chemokine and GPCR residue positions with paralog conservation scores ≥ 0.50 (detailed in Figure 2). The percentage of contacts among conserved chemokine and GPCR residue positions, versus contacts in which at least one of the two participating residues was non-conserved (i.e. paralog conservation ≤ 0.50), was calculated counting the number of contacts between conserved residues and dividing by the total number of contacts.

#### Analysis of CXCR4 saturation mutagenesis data

Heredia et al. assessed the functional effects of CXCR4 mutation on CXCL12 interactions by performing saturation mutagenesis of CXCR4 and assessing the ability of a CXCL12-GFP fusion protein to bind cells expressing single variant CXCR4 variants.^43^ Raw data from the paper supplement (https://journals.aai.org/jimmunol/article/200/11/3825/106401/Mapping-Interaction-Sites-on-Human-Chemokine/) were downloaded and manually “cleaned” by removing non-data-containing annotations, simplifying column headers, and labeling CXCR4 residue positions with CRN designations. Data provided in the supplement of that paper include log_2_ enrichment ratios for each variant that reflect the change in abundance of that variant after a selection process (i.e. fluorescent-activated cell sorting (FACS) for CXCL12-GFP-bound cells) relative to the same variant’s abundance prior to the selection process. Two replicates are provided in the data from Heredia et al. for each variant. We calculated the average value among both replicates for each substitution, then represented the range of substitution scores for all tested substitutions at a particular residue using a boxplot. Testing for significance between log_2_ enrichment values across various residue positions was calculated by Kruskal-Wallis test, with p-values determined by post-hoc Dunn test with Bonferroni correction for multiple comparisons. Statistical testing and graphs were done in R.

Saturation mutagenesis data were represented on the CXCL12-CXCR4 model^36^ by mapping the absolute value of mean log_2_ enrichment ratios across all substitutions and replicates for each position into the B-factor column of the associated PDB file. Absolute values of log_2_ enrichment ratios were used since mutation of functionally important positions from saturation mutagenesis experiments depletes these mutants after the selection step, such that functionally important positions are associated with negative log_2_ enrichment values, with more negative values corresponding to more important positions. Mapping was performed with use of “read.pdb” and “write.pdb” functions from the Bio3D package in R.^98^ Absolute values of mean per position log_2_ enrichment ratios were represented visually on the CXCL12-CXCR4 model by loading the modified PDB file into PyMol and representing the CXCR4 cartoon thickness using the “cartoon_putty” command, which represents larger absolute log_2_ enrichment values (i.e., functionally important) with a thicker radius. Log_2_ enrichment values were also represented using a color scale, with darker shades of grey indicating larger absolute log_2_ enrichment values. Identification of CXCL12 interaction “hotspots” on CXCR4 was done empirically by examination of CXCL12-CXCR4 complex structure with log_2_ enrichment ratios represented as putty and with color scale.

#### Subfamily-specific sequence and structural features

Contact comparison among CC versus CXC chemokine-GPCR complexes utilized the following structures and models: CCL2-CCR2 (7XA3),^29^ CCL3-CCR5 (7F1T),^30^ CCL5[5P7]-CCR5 (5UIW),^31^ CCL5[6P4]-CCR5 (7O7F),^32^ CCL5-CCR5 (7F1R),^30^ CCL15-CCR1 (7VL9),^28^ CCL20-CCR6 (6WWZ),^33^ CXCL8-CXCR1 (8IC0),^34^ CXCL8-CXCR2 (6LFO),^35^ CCL5-CCR5 (model),^31^ and CXCL12-CXCR4 (model).^36^ To devise a list of all possible contacts that contribute to CC- and CXC-subfamily specific interactions, subfamily scores for chemokine (y-axis) and receptor (x-axis) residues were plotted for each contact and filtered for contacts comprised of chemokine and GPCR residue positions with subfamily scores ≥75%.

Consensus CC- and CXC chemokine-GPCR contacts were identified by summating the CC and CXC chemokine-GPCR complexes that contain each contact. Since multiple versions of CCL5-CCR5 complexes were included in structural analysis (including two complexes with CCR5 bound to CCL5 N-terminal variants: [5P7]CCL5 (5UIW) and [6P4]CCL5 (7O7F), one model of CCL5-CCR5 based on the [5P7]CCL5 (5UIW)-CCR5 structure, and one native human CCL5-CCR5 complex containing incomplete structural resolution in key regions of the interface), we treated these complexes as a composite structure, such that any contact identified in any one of these complexes was considered a CCL5-CCR5 contact for the purposes of consensus CC and CXC-complex contact identification. With CCL5-CCR5 complexes considered as a composite complex, contacts were counted among five CC complexes (1: CCL2-CCR2 (7XA3), 2: CCL3-CCR5 (7F1T), 3: CCL5-CCR5 (5UIW, 7O7F, 7F1R, model from Zheng et al.^31^), 4: CCL15-CCR1 (7VL9), and 5: CCL20-CCR6 (6WWZ)) and three CXC complexes (1: CXCL8-CXCR1 (8IC0), 2: CXCL8-CXCR2 (6LFO), 3: CXCL12-CXCR4 (model from Ngo et al.^36^)). CC consensus contacts were defined as those contacts identified in a majority (i.e. ≥ 3/5) of CC complexes and no CXC contacts. CXC consensus contacts were defined as those contacts present in a majority of CXC complexes (i.e. ≥ 2/3) of CXC complexes and no CC complexes. This analysis yielded 15 consensus CC contacts and 6 consensus CXC contacts. Sequence logos were constructed for a subset of consensus CC and CXC contacts (Figure 3G) using human paralog CC and CXC chemokine/GPCR alignments, respectively via the R package “ggseqlogo”.^106^

#### Unique sequence and structural features of chemokine-GPCR complexes

##### Structurally preserved contacts among poorly conserved residues

To identify contacts that are (i) structurally preserved in a majority (i.e. > 8/16) of chemokine-GPCR complexes and (ii) comprised of poorly conserved chemokine and GPCR residues, the list of all 953 contacts was filtered for contacts occurring between chemokine and GPCR positions in which at least one of the contacting positions has paralog conservation score ≤ 0.5. For receptor positions, paralog conservation scores from conventional (non-atypical) receptor were used (see above). In total, 5 unique contacts were identified that were structurally preserved among > 8 chemokine-GPCR complexes (Figure S5C). The distribution of ortholog conservation scores (i.e. conservation of residue positions among 1:1 orthologs scored using Trident scoring system, see above) was represented for chemokine and GPCR positions contributing to these contacts using violin plots (Figure S5D). Plots represent ortholog conservation scores for the 43/46 chemokines for which 1:1 ortholog sequences were available and for 23/23 chemokine receptors for which 1:1 orthologs were available.

##### Pairwise comparisons of contacts and interface percent identity among all chemokine-GPCR complexes

Calculation of the percentage of contacts shared by each pair of human chemokine-GPCR complexes (i.e. CCL2-CCR2 (7XA3),^29^ CCL3-CCR5 (7F1T),^30^ CCL15-CCR1 (7VL9),^28^ CCL20-CCR6 (6WWZ),^33^ CXCL8-CXCR1 (8IC0),^34^ CXCL8-CXCR2 (6LFO),^35^ CXCL12-ACKR3 (7SK3),^37^ CX3CL1-CX3CR1 (7XBX),^38^ CCL5-CCR5 (model),^31^ and CXCL12-CXCR4 (model)^36^) was performed by counting the number of shared and unique contacts among each pair of complexes and calculating the percentage of contacts that are shared in both complexes relative to the total number of contacts (shared contacts plus unique contacts among each complex; Figure 4C). Calculation of the mean pairwise percent identity of interface residues was done by taking (1) the mean pairwise percent identity of chemokine positions at the chemokine interface (i.e., any position that makes a contact with a GPCR position among all 16 complexes), (2) the mean pairwise percent identity of receptor positions at the GPCR interface (i.e., any position that makes a contact with a chemokine position among all 16 complexes), and (3) taking the average of chemokine and receptor percent identities for a given pair of chemokine-GPCR complexes. Pairs of chemokine-GPCR complexes were colored according to (i) whether the chemokines or GPCRs comprising each pair of complexes have overlapping selectivity networks (lavender; e.g., CCL15-CCR1 and CCL3-CCR5 are considered overlapping since CCL15 and CCL3 both interact with CCR3); (ii) belong to the same subfamily but do not have overlapping networks (blue; e.g., CXCL12-CXCR4 and CXCL8-CXCR2 do not have overlapping networks but are comprised of CXC chemokines and receptors); or (iii) belong to the same family of chemokines and chemokine receptors but belong to different subfamilies (grey; e.g., CX3CL1-CX3CR1 and CCL20-CCR6).

##### Identification of chemokine and GPCR regions undergoing sequence- and structure-level diversification

To identify the chemokine and GPCR regions experiencing the most sequence- and structure-level differences among chemokine-GPCR complexes, we identified contacts from the 10 human chemokine-GPCR complexes and filtered for contacts that were (i) present in less than half (i.e., < 5/10) of these complexes and (ii) comprised of chemokine and GPCR residues with low paralog conservation (i.e., paralog conservation score < 0.5). Conventional (i.e., non-ACKR) paralog conservation scores were used for GPCRs. We then calculated the fraction of this subset of contacts made by each chemokine SSE, GPCR SSE, or paired chemokine-and-GPCR SSEs.

#### Enumeration of chemokine and GPCR fragments and identification of putative short linear motifs (SLiMs)

##### Selection of unstructured chemokine and receptor sequence segments

Chemokine N-termini, receptor N-termini, and receptor ECL2 were isolated from master chemokine (1058 sequences) and receptor (951 sequences) alignments. As described above, both unstructured regions were evaluated as insertion-free alignments by (1) removing insertions relative to the human sequence and (2) removing all alignment positions that correspond to gaps in the human sequence for each chemokine or receptor. This was done to maximize identification of functionally important motifs that are conserved among orthologs in intrinsically disordered regions, which are known to have elevated rates of insertions and deletions as compared to structured regions.^102,127^ Chemokine N-termini were defined as spanning from the first residue following the N-terminal signal peptide (determined from Uniprot) to the residue preceding CCN position CX.1. Chemokine receptor N-termini were defined as spanning from the first N-terminal residue to the residue preceding the first cysteine coming before TM1 in the GPCRdb alignment (i.e., the position preceding position Cys^1×22^ in this study). The endpoint of the CXCR6 N-terminus was His23, as CXCR6 is the only human receptor paralog lacking an N-terminal cysteine. Receptor ECL2 segments were defined as residues intervening and not including positions 4×65 and 5×31 in the GPCRdb alignment. Additionally, positions 45×50–45×52 are aligned in the GPCRdb alignment and removed from generation of sequence fragments.

##### Fragment generation, evaluation of fragment conservation, and identification of putative SLiMs

For chemokine and GPCR N-termini and GPCR ECL2, custom scripts were written to generate every 2-, 3-, and 4-mer peptide fragment for every sequence in the entire alignment. Degenerate (i.e. gapped) fragments were generated for 3- and 4-mers by introducing an “x” into the 2^nd^ position of all 3-mers (e.g., AxA) or into the 2^nd^, 3^rd^, or both positions of all 4-mers (e.g., AxAA, AAxA, AxxA). The functional properties of fragments from unstructured regions can be inferred from fragment conservation and the extent to which that fragment is shared among paralogous proteins.^128,129^ Following fragment generation, fragment conservation among orthologs was calculated by counting the number of times each fragment was represented among the number of orthologous sequences for that chemokine or receptor. For chemokines, because no 1:1 orthologs were identified for CCL4L1, XCL1, and XCL2, these chemokines were excluded from fragment analysis, and only 43 chemokines were considered for the calculation of conservation among chemokine paralogs. Fragments occurring multiple times in a single sequence were considered only once. For receptor ECL2 segments, fragments were generated separately for the segments preceding and following positions 45×50–52, and these lists were combined before counting the number of times each fragment was represented. As such, the same fragment occurring once before and after positions 45×50–52 would only be counted once for a particular receptor paralog. We draw a distinction between “fragments” described thus far and what we term “putative SLiMs” or simply “SLiMs” (we use “putative SLiMs” and “SLiMs” interchangeably). Fragments constitute any 2-, 3-, or 4-mer string of amino acids (including strings with gaps) enumerated from the sequences, whereas putative SLiMs constitute the subset of fragments that were identified in ≥ 50% ortholog sequences for that particular chemokine or receptor. In other words, putative SLiMs are conserved in ≥50% ortholog sequences for a particular chemokine/receptor, whereas fragments can be found in as few as one ortholog sequence for a particular chemokine/receptor. The conservation of all fragments among human paralogs was calculated by counting the number of chemokines or receptors sharing that particular fragment and dividing by the total number of chemokines or receptors. Fragment conservation scores were also generated for the conservation of each fragment among members of each chemokine/GPCR subfamily.

##### Generation of fragment fingerprints

For each unique peptide fragment belonging to chemokine or GPCR N-terminus or GPCR ECL2, we systematically recorded presence or absence among paralogs for each chemokine or receptor. We refer to the pattern of fragment presence/absence all chemokines or receptors as the *fragment fingerprint* (e.g., a 43-dimensional vector for chemokines; taking a value of 1 if present in that particular chemokine, or 0 if not). Fragment fingerprints describe whether a given fragment is unique or shared but not whether that particular fragment is conserved among orthologs (and this constitutes a candidate SLiM). Fragment fingerprints were clustered in Figure S6A by the extent to which they are shared, with fingerprints belonging to fragments found in a single chemokine (or receptor) at the top of the plot, and fingerprints belonging to fragments found in more than one chemokine (or receptors) at the bottom of the plot.

#### Natural variation and cancer mutation analysis

##### gnomAD natural variation data

Ensembl isoforms matching the sequences in Uniprot were identified and corresponding natural variation data were obtained from gnomAD release 2.1^52^ using custom written scripts. Gene-level, variant allele counts for variants occurring at interface positions (i.e., any chemokine or GPCR position that makes a contact in any of the 16 chemokine-GPCR complexes) were calculated by summing variant allele counts for any allele at an interface position for each chemokine and GPCR gene. The allele count metric is defined as the number of occurrences of a naturally occurring variant in the population of individuals queried. Variants occurring in individuals that are homozygous for a particular variant are counted twice, and a SNPs occurring in individuals that are heterozygous for a variant are counted once. Only missense variants (i.e., variants that cause a change in amino acid relative to the consensus sequence) were considered.

##### The Cancer Genome Atlas data and mutation calls

All The Cancer Genome Atlas (TCGA) data were retrieved from the GDC data portal (https://portal.gdc.cancer.gov/). For mutation calls, the MC3 call set (v0.2.8) was used, which integrates multiple computational approaches to call somatic mutations from over 400 TB of raw exome sequencing data collected from TCGA.^130^ In total, 10,295 tumors across 33 tumor lineages were used for analysis. For some genes, the mutations within the MC3 call set were provided for a different transcript than we had analyzed. For these genes, we mapped raw genomic locations from the MC3 call set to desired transcripts using ANNOVAR.^131^ General data processing and extraction were performed in MATLAB. Cancer-associated variants were calculated by summing the number of variants identified at any chemokine or GPCR interface position (i.e. any chemokine or GPCR position that makes a contact in any of the 16 chemokine-GPCR complexes) across chemokine and GPCR genes.

##### GeneATLAS genotype-phenotype data

For all chemokines and chemokine receptors, statistically significant genotype-phenotype associations were collected from the GeneAtlas database, which consists of pre-computed associations between 778 traits and 30 million variants, extracted from a cohort of 452,264 participants in the UK Biobank, on which exome sequencing was performed.^54^ Variants associated with a particular chemokine or receptor Ensembl gene ID (i.e. ENSG notation) were first retrieved from the Ensembl Variation database. Variants were then queried for disease associations in the GeneATLAS database. All associations of missense variants in coding regions with an association p-value less than 10^−8^ were extracted. For instances in which a chemokine or receptor had multiple Ensemble gene IDs, associated SNPs were retrieved for all gene IDs and pooled. Ensembl transcript IDs (i.e. ENST notation) were used to convert nucleotide-level SNPs associated with Ensembl gene IDs into specific residue positions. Since chemokine and receptor alignments incorporate a single, canonical sequence from Uniprot or sequence from OMA (see “Sequence acquisition” section), only SNPs associated with the corresponding Ensembl transcript IDs were considered.

##### Naturally occurring, cancer-associated, and phenotype-associated variant mapping to chemokine and chemokine receptor alignments

Naturally occurring, cancer-associated, and phenotype-associated variants were mapped to each position in chemokine and GPCR alignments using custom scripts. We note that protein sequences in both gnomad and TCGA are based on genomic sequences from Ensembl, whereas the alignment was based on sequences from Uniprot and OMA. As such there were a small number of instances in which there were mismatches between residues from gnomad and Uniprot/OMA sequences. In these instances, the Ensembl sequence was designated as the consensus sequence for variant calls.

#### vMIP-II residue analysis

To evaluate the extent to which vMIP-II residues resemble those of CC or CXC chemokines at the same positions, all residue positions in the vMIP-II sequence were evaluated using position-specific logistic regression models trained on CC and CXC sequences as follows. First, a logistic regression model was trained using a single sequence position from an equivalent number of CC and CXC sequences (described above). Second, the trained model was “tested” on the corresponding position from the vMIP-II sequence to generate a *prediction probability score* indicating the likelihood that the position belongs to a CC or a CXC chemokine. A score closer to 0 suggests with high confidence that the particular position belongs to a CC receptor, and a score closer to 1 suggests with high confidence that the particular position belongs to a CXC receptor. Third, scores were generated in triplicate by using a random seed to partition CC and CXC sequences into the training and test set (see above section for details). Fourth, the mean prediction probability score from all three replicates was taken per position for each vMIP-II residue position. Beyond the vMIP-II sequence, per position prediction probability scores were generated in triplicate as described for other human chemokine sequences.

The distribution of vMIP-II prediction probability scores was plotted for all positions of vMIP-II and a set of human CC and CXC chemokines which are part of chemokine-GPCR complex structures or models. Mean prediction probability scores were analyzed for all residues with subfamily scores ≥ 75% (i.e. only for positions at which CC-versus-CXC predictions were considered accurate, see above). The distribution of mean prediction probability scores for subfamily-predictive positions were then plotted for (i) all chemokine sequence positions meeting this criterion (Figure S7F) and (ii) chemokine sequence positions meeting this criterion that also make intermolecular contacts with GPCRs as part of a complex (Figure 6C). For the latter, chemokine contacts were derived from contact analysis CCL2-CCR2 (7XA3), CCL3-CCR5 (7F1T), CCL5-CCR5 (model from Zheng et al.^31^), CCL15-CCR1 (7VL9), CCL20-CCR6 (6WWZ), CXCL12-CXCR1 (model from Ngo et al.^36^), CXCL8-CXCR2 (6LFO), and vMIP-II-CXCR4 (4RWS). Distribution plots were made using R package “ggridges” (https://wilkelab.org/ggridges/).^107^

Percentages of contacts made by CC-versus-CXC-like residue positions from CCL5-CCR5 (model from Zheng et al.^31^) versus vMIP-II-CXCR4 (4RWS) were calculated by counting the number of contacts made by residues with prediction probability scores > 0.5 (i.e. CXC-like residue positions) and < 0.5 (i.e. CC-like residue positions) among positions with subfamily scores > 75% and dividing by the total number of contacts made by members of both groups. Prediction probability scores were represented for the same CC- and CXC-like positions on the chemokines from the respective complexes by mapping them to the B-factor column and representing them using the “cartoon_putty” command in Pymol as well as mapping to represent CC-like positions with red and CXC-like positions in blue, with increased color intensity reflecting scores closer to 1.0 and 0.0, respectively.

Representation of the change in prediction probability scores by mutating vMIP-II Lys10Thr^NTc.CM1^ and Arg7Ile^NTc.Cm4^ were made by plotting mean prediction probability scores for WT vMIP-II and CCL8 (which bears the same residues at those to which vMIP-II is mutated) on the same plot.

#### β-arrestin recruitment assays

Chemokine-induced β-arrestin recruitment to WT and mutated receptors was measured by Nanoluciferase complementation assay (NanoBiT, Promega Corporation, Madison, WI, USA).^45,132–134^ Briefly, 5 × 10^6^ HEK293T cells were seeded in 10-cm culture dishes and 24 h later co-transfected with pNBe vectors encoding human chemokine receptors C-terminally fused to SmBiT and β-arrestin-1 N-terminally fused to LgBiT. 24 h after transfection cells were harvested, incubated for 15 minutes at 37°C with 200-fold diluted Nano-Glo Live Cell substrate, and distributed into white 96-well plates (5 × 10^4^ cells per well). In agonist mode, cells were then incubated with chemokines at concentrations ranging from 0.03 nM to 1 μM. In antagonist mode, cells were incubated with the antagonist chemokine (vMIP-II) at concentrations ranging from 0.03 nM to 1 μM in the presence of agonist chemokine at a concentration corresponding to EC_80_ value (5 nM CCL5 on CCR5 and 20 nM CXCL12 on CXCR4). β-arrestin recruitment to receptors was evaluated by measuring bioluminescence with a Mithras LB940 luminometer (Berthold Technologies). Concentration–response curves were fitted to the three-parameter Hill equation using an iterative, least-squares method (GraphPad Prism version 10.0.0). All curves were fitted to data points generated from the mean of at least three independent experiments. All chemokines were provided by Protein Foundry, LLC.

#### Chemokine binding assays

Ligand binding to ACKR1 Gly42 and Asp42 variants was monitored by NanoBRET. Briefly, 5 × 10^6^ HEK293T cells were plated in 10-cm culture dishes and 24 h later transfected with vectors encoding receptor variants N-terminally fused to Nanoluciferase. 24 h after transfection, cells were harvested and distributed into white 96-well plates (1 × 10^5^ cells per well). Cells were then incubated 2 h on ice with AZDye 488-labelled chemokines (Protein Foundry) at concentrations ranging from 0.1 nM to 1 mM. Coelenterazine H (diluted 1:500) was then added and donor emission (450/8nm BP filter) and acceptor emission (530nm LP filter) were immediately measured on a GloMax Discover plate reader (Promega). Concentration–response curves were fitted to the three-parameter Hill equation using an iterative, least-squares method (GraphPad Prism version 10.0.0). All curves were fitted to data generated from the mean of at least three independent experiments. All chemokines were provided by Protein Foundry, LLC.

#### Calcium flux assays

Ready-to-Assay Chem-1 cells (Eurofins) expressing human CCR10 or human CCR3 were prepared to assay according to the manufacturer’s instructions. Briefly, cell vials were thawed, washed, and re-suspended in Media Component prior to being seeded into a 96-well plate. Cells were incubated at 37°C, 5% CO_2_ for 24 hours. Following the 24-hour incubation period, calcium flux assays were performed as previously described.^135^ Briefly, the media was removed, and the cells were washed 1x with 100μL Ca^2+^ and Mg^2+^-free HBSS (Life Technologies). Subsequently, 100μL calcium flux cell buffer (Ca^2+^/Mg^2+^-free HBSS supplemented with 20mM HEPES and 0.1% bovine serum albumin) and 100mL FLIPR Calcium 4 or Calcium 6 Dye (Molecular Devices) were added to each well, such that the ratio of dye to cell buffer was 1:1. Plates were then centrifuged for <1 minute at 1000 × g to ensure that all cells were settled on the bottom of the wells. Plates were incubated for 45 minutes at 37°C, 5% CO_2_. Fluorescence was measured at 37°C using a FlexStation 3 Microplate Reader (Molecular Devices) with excitation and emission wavelengths at 485 and 515nm, respectively. After an 18 second baseline measurement, the indicated concentrations chemokine were added, and the resulting calcium response was measured for an additional 82 seconds. Fluorescence as a function of chemokine concentration was fitted to four-parameter equation (GraphPad Prism). Experiments were recorded in triplicate on three separate days. The averages from each experiment were normalized to the maximal response of WT-CCL28 (100% activity). WT CCL28 and variants were expressed and purified from *E. coli* following previously established protocols.^95^

#### Isothermal titration calorimetry assays

ACKR1 peptide construct (ACKR1^term^) includes the N-terminal residues 1–60 with alanine substitution of cysteines. ACKR1^Nterm^ was recombinantly expressed in *E. coli*, purified, and isolated with reverse-phase chromatography using the protocol published in Gutjahr et al.^77^ The eluted ACKR1^N-term^ product was lyophilized, and its identity confirmed by linear ion trap quadrupole mass spectrometry (LTQ-MS). CXCL12 chemokine was purified using the protocol published in Veldkamp et al.^136^ and identity confirmed by LTQ-MS. Isothermal titration calorimetry (ITC) data were collected on a Microcal VP-ITC. CXCL12 and ACKR1^N-term^ proteins were both dialyzed in 2,000 MWCO Slide-A-Lyzer (Thermo-Fisher) mini dialysis units against dialysis buffer of 20 mM MES at pH 6.5. CXCL12 solution prepared to 20 μM and ACKR1^N-term^ to 200 μM using dialysis buffer. ITC cell was loaded with chemokine solution and titrated by injecting 10 μL of ACKR1^N-term^ with a 210- second spacing, a reference power of 10 μcal/sec, stirring of 307 rpm, and a temperature set at 26 °C.

#### Chemotaxis experiments

##### Cell lines and healthy donor T cells

To evaluate CXCR4-mediated cell migration, primary human T cells were isolated from human PBMCs (see “experimental model” section above). For generation of ZsGreen-labeled T cells, enriched T cells were thawed and resuspended (see above) and stimulated overnight on 24-well non-tissue-culture treated plates that were precoated with CD3 and CD28 antibodies (Miltenyi). A lentiviral vector expressing ZsGreen was generated using the pCL45 backbone as previously described,^137^ with insulators removed from the self-inactivating 3’ partially deleted viral LTRs based on the safety records of LVs in clinical trials.^138^ The ZsGreen expression cassette is under the control of the MND promotor and was cloned by standard InFusion techniques. The final cloned vector was verified by sequencing (Hartwell Center, St. Jude Children’s Research Hospital, Memphis, TN, USA). Transduction was performed on day 0 by adding ZsGreen-containing LV particles at an MOI of 50 TU/cell and protamine sulfate at 4 mg/mL after empiric determination of adequate MOI. On day 3, T cells were transferred into new 24-well tissue culture treated plates and subsequently expanded with IL7 and IL15 (10 ng/mL each). Each donor was counted on day 3 and day 5 post transduction. Cells were used for experiments between days 7 and 10 post transduction. All cell culture was maintained at 37⁰C in 5% CO_2_ and 95% humidity in standard laboratory incubators.

##### Flow cytometry

CD4, CD8, CCR7, CD45RO, CXCR4, CCR5, and ZsGreen expression on cells was evaluated using flow cytometry. A FACSCanto II (BD Biosciences) instrument was used to acquire flow cytometry data, which was analyzed using FlowJo v10 (BDBiosciences). For surface staining, samples were washed with and stained in PBS (Lonza) with 1% FBS. For all experiments, known negatives (e.g., nontransduced T cells) served as gating controls. Cells were stained with fluorochrome-conjugated antibodies using CXCR4 (clone 12g5, APC-conjugated, Biolegend), CCR5 (clone J418F1, APC-conjugated, Biolegend), and CCR3 (clone 5E8, APC-conjugated, Biolegend) antibodies as appropriate. Where applicable, ZsGreen signal as assessed by flow cytometry was used to assess transduction efficiency. For phenotyping, T cells were stained with fluorochrome-conjugated antibodies using combinations of the following markers: CD4 (clone SK3, BD Biosciences), CD8 (clone SK1, BD Biosciences), CCR7 (clone G043H7, BioLegend, San Diego, CA, USA), and CD45RO (clone UCHL1, BD Biosciences). Cells were additionally stained with DAPI (BD Biosciences) to gate for live cells.

##### vMIP-II proteins and chemokines

Recombinant human vMIP-II, vMIP-II Lys10Thr, vMIP-II Arg7Ile, vMIP-II Leu13Phe, and vMIP-II Lys10Thr/Arg7Ile/Leu13Phe were synthesized by Protein Foundry (Milwaukee, WI) and resuspended according to manufacturer’s instructions. For assays evaluating CXCR4-mediated chemotaxis, recombinant human CXCL12 at 50 ng/mL in solution (Protein Foundry) was utilized as a chemoattractant, and for assays evaluating CCR5-mediated chemotaxis, recombinant human CCL5 at 50 ng/mL in solution (Protein Foundry) was utilized as a chemoattractant.

##### Semihalo migration assay

To evaluate small differences in migration of human leukocytes secondary to vMIP-II protein and mutant vMIP-II protein inhibition of canonical chemokine axis signaling, the semihalo migration assay was developed, which allows detailed and kinetic visualization of migration of cells along a chemokine gradient. Briefly, the target chemokine attractant is plated within a Matrigel (Corning, Glendale, AZ, USA) matrix in a semihalo along the right edge of a 96-well plate well, and the cells of interest are plated within a similar Matrigel matrix along the left edge of the same well. After matrix solidification, appropriate media is added to the well, and the plate is placed within the Incucyte live cell microscopy system. As the chemokine leeches out of the semihalo matrix on the right side of the well, the cells within the left sided matrix migrate along the resulting gradient from left to right.

##### CXCR4/CXCL12-mediated migration of healthy donor T cells

Recombinant human CXCL12 (Protein Foundry) was obtained and reconstituted at 1000 μg/mL in deionized water with 0.1% BSA. A 4:3 preparation of Matrigel:serum-free RPMI with 1% GlutaMAX was prepared sufficient to plate 5 μL per desired sample well. For those wells containing CXCL12 as a chemoattractant, CXCL12 was added to the Matrigel preparation at a final concentration of 1.5 ng/μL. This dose was chosen to create a final concentration within the well of 50 ng/mL once all CXCL12 had leeched from the chemoattractant semihalo matrix into the desired 150 μL of media within the well. Five μL of Matrigel suspension only or Matrigel-suspended rhCXCR4 was then plated in a semi-halo configuration using an E3X Repeater^®^ pipette (Eppendorf) with a 0.1 mL Combitip advanced pipette tip along the right side of each 96 well plate well. Plate was kept tilted at 30° angle for 10 mins as the Matrigel matrix was allowed to solidify.

T cells pre-incubated with serum-free media and experimental vMIP-II proteins were then pelleted and the media aspirated. Cells were resuspended in 4:3 Matrigel:serum-free RPMI preparation at a concentration of 2 × 10^5^ cells per 5 μL of Matrigel preparation. The cells were then plated using the E3X Repeater^®^ pipette with a 0.1 mL Combitip advanced pipette tip at a volume of 5 μL per experimental well in a semihalo configuration along the left side of the experimental well. The plate was kept tilted at a 30° angle to facilitate solidification of the Matrigel in the semihalo configuration for 10 minutes.

After both semihalos were plated and solidified, 150 μL of serum-free RPMI with 1% GlutaMAX was added to each well taking care not to disturb the semihalos. The plate was then placed on the Incucyte cradle and the assay initiated.

##### Data analysis

For both healthy donor T cells, phase and green fluorescent images were acquired on the Incucyte system at intervals of 1 hour. Background signal in the green fluorescent channel was subtracted using TopHat segmentation. Migration was quantified as the area of detected green signal per well (healthy donor T cells) in μm^3^ at each time point. For analysis, each time point had the starting area of signal in each well subtracted so that only additional migrated cell area above baseline within the well was counted as active migration.

### QUANTIFICATION AND STATISTICAL ANALYSIS

Statistical parameters are reported in figures, figure legends, text, and method details including replicate numbers, p-values, and statistical tests used where appropriate. In brief, all functional data shown in Figures and Tables were presented as the mean ± SEM or SD (as indicated in the associated legend) unless otherwise indicated, with the ‘N’ values indicating the numbers of independent experiments. For comparison of functional impact of residue mutations using CXCR4 saturation mutagenesis data, data were analyzed in R (see method details, above), and statistical testing was done by Kruskal-Wallis test with p-values determined by post-hoc Dunn test with Bonferroni correction for multiple comparisons. For analysis of whether CC versus CXC chemokines are more likely to couple receptors of their same subfamily, experimentally validated interactions among ‘like’ (e.g., CC chemokine-to-CC receptor) and ‘unlike’ (e.g., CC chemokine-to-CXC receptor) chemokines and GPCRs were counted to create a contingency table, and a p-value was calculated using a Chi-squared test to evaluate for the significance of subfamily identity and interaction partner. The analysis was done in R. For CCR5 and CXCR4 β-arrestin recruitment assays and ACKR1 fluorescence-based binding assays, concentration-response curves were fitted to the three-parameter Hill equation using an iterative, least-squares method using GraphPad Prism. All curves were fitted to data points generated from the mean of at least three independent experiments. For CCR3 and CCR10 calcium flux experiments, fluorescence as a function of chemokine concentration was fitted to a four-parameter equation using GraphPad Prism, and curves were fitted from a mean of three independent experiments. For the ACKR1 ITC experiments, isotherm data were fit using Origin 7.0 software (OriginLab) using the one-site binding model. Average measurements from ITC isotherms (*n* = 2) are shown ± the mean absolute error, except the calculated ΔS, which are shown as ± the standard deviation. For comparison of ortholog conservation of fragments identified in the ACKR1 N-terminus, ACKR1 fragments themselves were excluded to selectively assess conservation and infer the function of these fragments more broadly among the chemokine receptor family. A p-value was calculated by Wilcoxson test. For chemotaxis experiments, for healthy human donor-derived T cells, a minimum of three healthy donors were utilized to account for inter-donor variability in cell health and migration capability, with three technical replicates per donor.

## Supplementary Material

MMC1

MMC2

MMC3

MMC4

MMC5

1

Supplemental information can be found online at https://doi.org/10.1016/j.cell.2025.03.046.

## Figures and Tables

**Table:** KEY RESOURCES TABLE

REAGENT or RESOURCE	SOURCE	IDENTIFIER
Antibodies		

Immunocult CD3/CD28 T cell activator	Stem Cell	10971; RRID: AB_2827806
Anti-CXCR4-APC; clone 12g5	Biolegend	306510; RRID: AB_314616
Anti-CCR5-APC; clone J418F1	Biolegend	359122; RRID: AB_2564073
Anti-CCR3-APC; clone 5E8	Biolegend	310708; RRID: AB_2228976
Anti-CD4-PE-Cy7; clone SK3	BD Biosciences	557852; RRID: AB_396897
Anti-CD8-APC-H7; clone SK1	BD Biosciences	560179; RRID: AB_1645481
Anti-CCR7-APC; clone G143H7	Biolegend	353214; RRID: AB_10917387
Anti-CD45RO-PerCP-Cy5; clone UCHL1	BD Biosciences	560607; RRID: AB_1727500

Bacterial and Virus Strains		

Escherichia coli strain SG13009 [pRPEP4]	Qiagen	https://www.qiagen.com/us/resources/resourcedetail?id=abc8b958-d415-4b91-88a1-e5f90d0a1884&lang=en

Chemicals, Peptides, and Recombinant Proteins		

CCL3	Protein Foundry	https://www.proteinfoundry.com
CCL4	Protein Foundry	https://www.proteinfoundry.com
CCL5	Protein Foundry	https://www.proteinfoundry.com
CXCL12	Protein Foundry	https://www.proteinfoundry.com
AZDye 488-CCL2	Protein Foundry	https://www.proteinfoundry.com
AZDye 488-CCL7	Protein Foundry	https://www.proteinfoundry.com
AZDye 488-CXCL1	Protein Foundry	https://www.proteinfoundry.com
AZDye 488-CXCL8	Protein Foundry	https://www.proteinfoundry.com
AZDye 488-CXCL11	Protein Foundry	https://www.proteinfoundry.com
AZDye 488-CXCL12	Protein Foundry	https://www.proteinfoundry.com
IL-7	PeproTech	200-07
IL-15	PeproTech	200-15
vMIP-II	Protein Foundry	https://www.proteinfoundry.com
vMIP-II R7I	Protein Foundry	https://www.proteinfoundry.com
vMIP-II K10T	Protein Foundry	https://www.proteinfoundry.com
vMIP-II L13F	Protein Foundry	https://www.proteinfoundry.com
vMIP-II R7I, K10T, L13F	Protein Foundry	https://www.proteinfoundry.com
CXCL12	Protein Foundry	https://www.proteinfoundry.com
CCL5	Protein Foundry	https://www.proteinfoundry.com
ACKR1 1-60, C4A, C51A, C54A	Gutjahr et al.^77^	N/A
ACKR1 1-60, C4A, G42D, C51A, C54A	This manuscript	N/A
FLIPR Calcium 4 and Calcium 6 Dyes	Molecular Devices	https://www.moleculardevices.com/products/assay-kits/gpcrs/flipr-calcium-assay-kits
HBSS, no Ca^2+^, no Mg^2+^	Life Technologies	14170112
Coelenterazine H	Promega	S2011
Nano-Glo live Cell substrate	Promega	https://www.promega.com/products/luciferase-assays/reporter-assays/nano-glo-extended-live-cellsubstrates/?catNum=N2570

Critical Commercial Assays		

Ready-to-Assay^™^ CCR3 Chemokine Receptor Frozen Cells	Eurofins	HTS008RTA
Ready-to-Assay^™^ CCR10 Chemokine Receptor Frozen Cells	Eurofins	HTS014RTA
NanoBiT PPI Assay system	Promega	https://www.promega.com/products/protein-interactions/live-cell-protein-interactions/nanobit-ppi-starter-systems/?catNum=N2014&accordion0=2,3

Deposited Data		

Solution structure of the human CC chemokine, I-309	Protein Data Bank	PDB: 1EL0
Monocyte chemoattractant protein 1, P-form	Protein Data Bank	PDB: 1DOK
The crystallographic structure of the complex between Evasin-1 and CCL3	Protein Data Bank	PDB: 3FPU
Solution structure of the monomeric variant of the chemokine MIP-1beta	Protein Data Bank	PDB: 1JE4
Crystal Structure of human RANTES mutant K45E	Protein Data Bank	PDB: 1U4P
Determination CC-chemokine MCP-3, NMR, 7 structures	Protein Data Bank	PDB: 1NCV
Crystal structure of human Monocyte Chemotactic Protein-2	Protein Data Bank	PDB: 1ESR
Solution NMR structure of Eotaxin, minimized average structure	Protein Data Bank	PDB: 1EOT
Crystal Structure of Human Monocyte Chemoattractant Protein 4 (MCP-4/CCL13)	Protein Data Bank	PDB: 2RA4
Structural and Functional Characterization of CC Chemokine CCL14	Protein Data Bank	PDB: 2Q8R
Solution structure of the human chemokine HCC-2, NMR, 30 structures	Protein Data Bank	PDB: 2HCC
Structure of human chemokine CCL16	Protein Data Bank	PDB: 5LTL
High resolution crystal structures of thymus and activation-regulated chemokine	Protein Data Bank	PDB: 1NR4
Crystal structure of CC-chemokine 18	Protein Data Bank	PDB: 4MHE
Solution structure of the human chemokine CCL19	Protein Data Bank	PDB: 2MP1
Human MIP-3alpha/CCL20	Protein Data Bank	PDB: 1M8A
Crystal Structure of Truncated CCL21	Protein Data Bank	PDB: 5EKI
Solution structure of myeloid progenitor inhibitory factor-1 (MPIF-1)	Protein Data Bank	PDB: 1G91
Solution structure of the human chemokine Eotaxin-2	Protein Data Bank	PDB: 1EIG
Solution structure of Eotaxin-3	Protein Data Bank	PDB: 1G2S
Solution structure of the human chemokine CCL27	Protein Data Bank	PDB: 2KUM
The NMR solution structure of CCL28	Protein Data Bank	PDB: 6CWS
Solution structure of GRO/melanoma growth stimulatory activity determined by 1H NMR spectroscopy	Protein Data Bank	PDB: 1MSG
Truncated human GROB[5-73], NMR, 20 structures	Protein Data Bank	PDB: 1QNK
Crystal structure of recombinant human platelet factor 4	Protein Data Bank	PDB: 1RHP
Crystal Structure of CXCL4L1	Protein Data Bank	PDB: 4HSV
Solution structure of CXCL5	Protein Data Bank	PDB: 2MGS
The crystal structure of recombinant human neutrophil-activating peptide-2 (M6L) at 1.9-angstroms resolution	Protein Data Bank	PDB: 1NAP
The atomic resolution crystal structure of human IL-8	Protein Data Bank	PDB: 5D14
CXCR3 Binding Chemokine IP-10/CXCL10	Protein Data Bank	PDB: 1LV9
NMR Structure of CXC Chemokine CXCL11/ITAC	Protein Data Bank	PDB: 1RJT
Crystal Structure of recombinant Human Stromal Cell-Derived Factor-1alpha	Protein Data Bank	PDB: 2J7Z
Crystal structure of CXCL13	Protein Data Bank	PDB: 4ZAI
Solution structure of Brak/CXCL14	Protein Data Bank	PDB: 2HDL
Solution structure of human lymphotactin	Protein Data Bank	PDB: 1J9O
CCL2-CCR2 complex structure	Shao et al.^29^; RCSB/PDB	PDB: 7XA3
CCL3-CCR5 complex structure	Zhang et al.^30^; RCSB/PDB	PDB: 7F1T
CCL5[5P7]-CCR5 complex structure	Zheng et al.^31^; RCSB/PDB	PDB: 5UIW
CCL5[6P4]-CCR5 complex structure	Isaikina et al.^32^; RCSB/PDB	PDB: 7O7F
CCL5-CCR5 complex structure	Zhang et al.^30^; RCSB/PDB	PDB: 7F1R
CCL15-CCR1 complex structure	Shao et al.^28^; RCSB/PDB	PDB: 7VL9
CCL20-CCR6 complex structure	Wasilko et al.^33^; RCSB/PDB	PDB: 6WWZ
CXCL8-CXCR1 complex structure	Ishimoto et al.^34^; RCSB/PDB	PDB: 8IC0
CXCL8-CXCR2 complex structure	Liu et al.^35^; RCSB/PDB	PDB: 6LFO
CXCL12-ACKR3 complex structure	Yen et al.^37^; RCSB/PDB	PDB: 7SK3
CX3CL1-CX3CR1 complex structure	Lu et al.^38^; RCSB/PDB	PDB: 7XBX
vMIP-II-CXCR4 complex structure	Qin et al.^39^; RCSB/PDB	PDB: 4RWS
CX3CL1-US28 complex structure	Burg et al.^40^; RCSB/PDB	PDB: 4XT1
CX3CL1.35-US28 complex structure	Miles et al.^41^; RCSB/PDB	PDB: 5WB2
CCL5-CCR5 complex model	Zheng et al.^31^	https://www.cell.com/immunity/fulltext/S1074-7613(17)30218-2?_returnURL=https%3A%2F%2Flinkinghub.elsevier.com%2Fretrieve%2Fpii%2FS1074761317302182%3Fshowall%3Dtrue#supplementaryMaterial; (“Document S2”)
CXCL12-CXCR4 complex model	Ngo et al.^36^	https://journals.plos.org/plosbiology/article?id=10.1371/journal.pbio. 3000656#sec020 (“S2 Data”)
Ensembl	Aken et al.^91^	https://www.ensembl.org/index.html
GeneATLAS	Canela-Xandri et al.^54^	http://geneatlas.roslin.ed.ac.uk
gnomAD Genome Aggregation Database	Karczewski et al.^52^	https://gnomad.broadinstitute.org
GPCRdb	Pandy-Szekeres et al.^92^	https://gpcrdb.org
OMA Orthology Database	Altenhoff et al.^93^	https://omabrowser.org/oma/home/
The Cancer Genome Atlas	Cancer Genome Atlas Research^94^	https://portal.gdc.cancer.gov

Experimental Models: Cell Lines		

Ready-to-Assay^™^ CCR3 Chemokine Receptor Frozen Cells	Eurofins	HTS008RTA
Ready-to-Assay^™^ CCR10 Chemokine Receptor Frozen Cells	Eurofins	HTS014RTA
HEK293T cells	Abcam	ab255449
De-identified human donor PBMCs	Primary donor	N/A

Recombinant DNA		

CCR5 vectors (WT and mutants)	This paper	N/A
CXCR4 vectors (WT and mutants)	This paper	N/A
ACKR1 vectors (WT and mutants)	This paper	N/A
β-arrestin-1 vectors	This paper	Described in PMID: 35623707
pQE30-ACKR1 (1-60/C4A/C51A/C54A)	Gutjahr et al.^77^	N/A
pQE30-ACKR1 (1-60/C4A/G42D/C51A/C54A)	This manuscript	N/A
pET28a-CCL28	Thomas et al.^95^	N/A
pET28a-CCL28 (ΔS)	This paper	N/A
pET28a-CCL28 (ΔSE)	This paper	N/A
pET28a-CCL28 (ΔSEA)	This paper	N/A
pET28a-CCL28 (ΔSEAI)	This paper	N/A
pCL45.MND.P2A.ZsGreen	This manuscript	N/A
GFP-FFLUC	Hebbar et al.^96^	N/A

Software and Algorithms		

Adobe Illustrator	N/A	https://www.adobe.com/products/illustrator.html
ANNOVAR	Wang et al.^97^	http://annovar.openbioinformatics.org/en/latest/
Bio3d	Grant et al.^98^	http://thegrantlab.org/bio3d/
BD FACSDiva v 8.0	BD Biosciences	N/A
Clustal Omega	Sievers et al.^99^	www.ebi.ac.uk/Tools/msa/clustalo/
Cytoscape version 3.7.1	Shannon et al.^100^	https://cytoscape.org
FlowJo v10	BD Biosciences	N/A
GraphPad Prism	GraphPad Software	https://www.graphpad.com
Incucyte 2022A	Sartorius	N/A
Jalview	Waterhouse et al.^101^	https://www.jalview.org/
KMAD algorithm	Lange et al.^102^	https://www3.cmbi.umcn.nl/kmad/about/
MATLAB	MathWorks	https://www.mathworks.com/products/matlab.html
MstatX (trident scoring algorithm)	Valdar^103^	https://github.com/gcollet/MstatX
MUSCLE	Edgar^104^	https://www.ebi.ac.uk/Tools/msa/muscle/
MUSTANG	Konagurthu et al.^105^	http://lcb.infotech.monash.edu.au/mustang/
Protein Contact Atlas	Kayikci et al.^42^	https://www.mrc-lmb.cam.ac.uk/pca/index.html
PyMOL	Schrödinger	https://pymol.org/2/
R	R Core Team	https://cran.r-project.org
R package ggseqlogo	Wagih^106^	https://cran.r-project.org/web/packages/ggseqlogo/index.html
R package ggridges	Wilke^107^	(https://wilkelab.org/ggridges/)

Other		

Chemokine-GPCR web resource	This paper	https://andrewbkleist.github.io/chemokine_gpcr_encoding/
AutoMACS Pro separator	Miltenyi	N/A
FACS Canto II	BD Biosciences	N/A
Incucyte	Sartorius	N/A
CD4+ Microbeads	Miltenyi	120000440
CD8+ Microbeads	Miltenyi	130045201
AutoMACS Pro columns	Miltenyi	130021101
Recovery ^™^ Cell culture media	Gibco	12648-010
RPMI1640	Cytiva	SH30096.01
10% FBS	GE Healthcare	N/A
1% GlutaMAX	Gibco	35050-061
Protamine sulfate		N/A
PBS	Corning	21-031-CV
Reduced-growth factor Matrigel	Corning	CB-40230
MACS BSA Stock solution	Miltenyi	130-091-376
E3X Repeater ^®^ Pipette	Eppendorf	4987000118
Combitip 0.1 ml pipette tip	Eppendorf	30089510
Tissue culture flasks, T175	Corning	353112
Tissue culture flasks, T75	TRP	90076
6 well cell culture dish	Falcon	353046
12 well cell culture dish	Corning	3513
24 well cell culture dish	TRP	92024
24 well non-tissue culture treated cell culture dish	MidSci	667524
96 well cell culture dish	Corning	3599

## Highlights

- Conserved and variable selectivity determinants drive chemokine-GPCR interactions
- Selectivity and promiscuity emerge from unstructured and structured protein regions
- We engineer a viral chemokine with altered GPCR preference using selectivity principles
- Web resource for sequence-structure-function studies and protein design efforts
