# Supplementary material for: Encoding and decoding selectivity and promiscuity in the human chemokine-GPCR interaction network
Source: Cell. Author manuscript; Available in PMC 2025 Sep 15. (PMC12435897; doi:10.1016/j.cell.2025.03.046)
Supplement: 1 [file NIHMS2076408-supplement-1.pdf]

CellPress
OPEN ACCESS

Supplemental figures

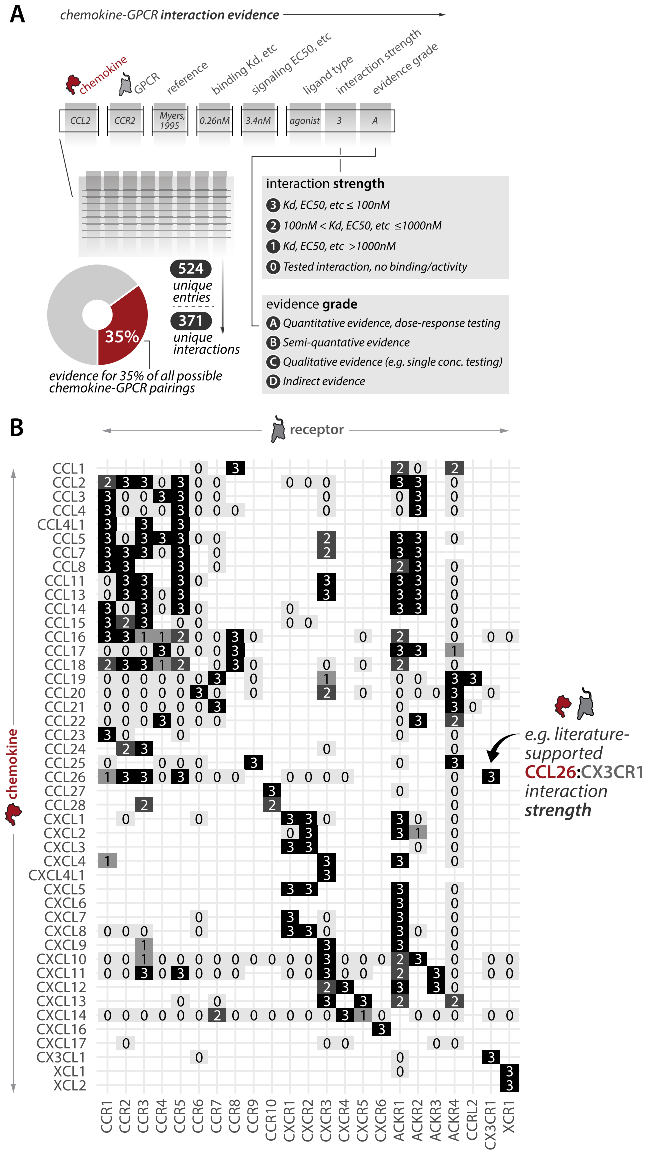

Figure S1. Literature-supported chemokine-GPCR interaction network, related to Figure 1

(A) Overview of approach to grading interaction strength for chemokine-GPCR pairings and devising evidence grades for documented interactions.

(B) Matrix representing all chemokine-GPCR interactions for which interaction strength could be assigned (includes all evidence grades). For interactions for which different evidence grades were assigned (based on different literature sources), the maximum evidence strength is shown. See also Table S1 and STAR Methods.

A

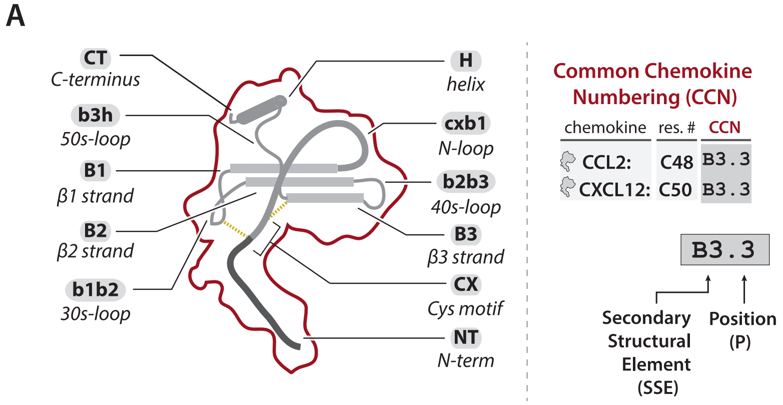

B

SSE	Classical Name	Length of consensus domain
NT	N-term	NA
CX	CC, CXC motif	5
cxb1	N-loop	19
B1	β1-strand	7
b1b2	30s-loop	25
B2	β2-strand	6
b2b3	40s-loop	12
B3	β3-strand	4
b3h	50s-loop	6
H	helix	10
CT	C-term	NA

C

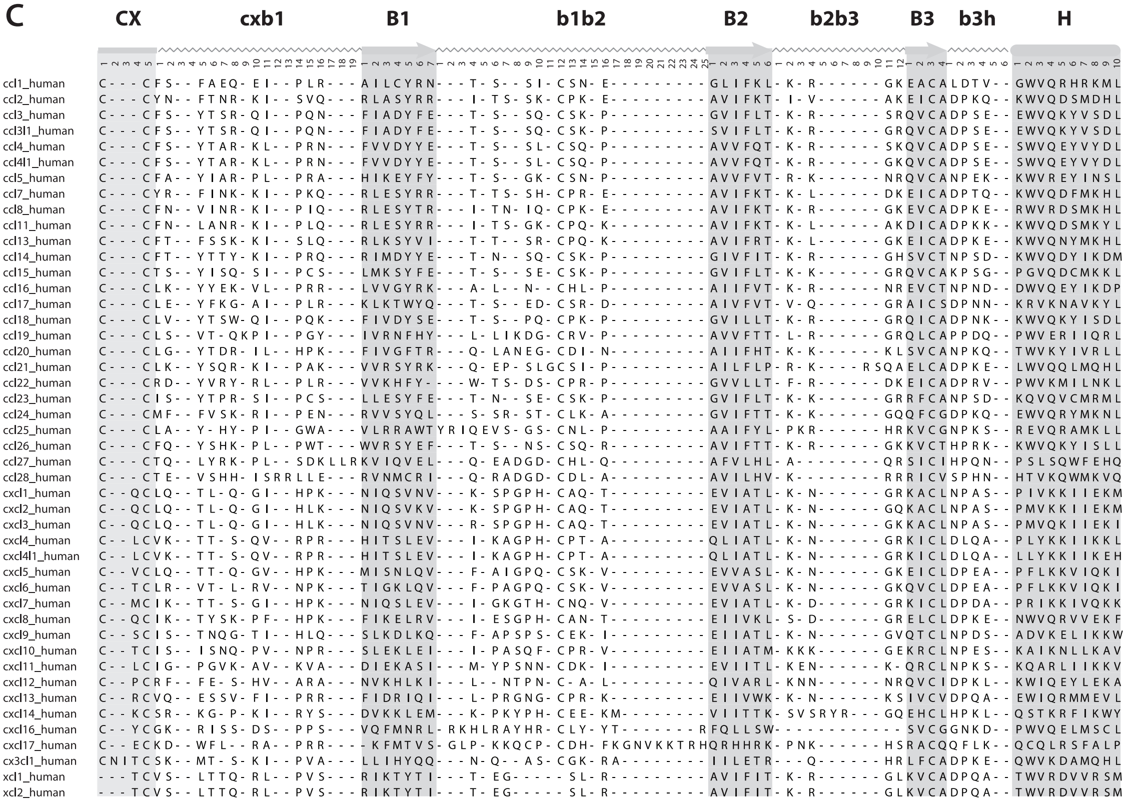

Figure S2. CCN, related to Figure 2

(A) Common chemokine numbering (CCN). Each secondary structural element (SSE) of the chemokine is given a designation (e.g., CX for Cys motif, B1 for β1 strand, etc.), and each residue within an SSE is given a positional index P (e.g., B1.1 [SSE.P] is the first residue within the β1 strand). This system allows comparison of structurally equivalent positions across different chemokines. For instance, position B3.3 corresponds to C48 in CCL2 and C50 in CXCL12 due to differences in the length of their respective N termini.

(B) The number of residue positions and classical names are listed for each CCN SSE.

(C) Alignment of all 46 human paralogs with CCN SSEs and positions indicated above.

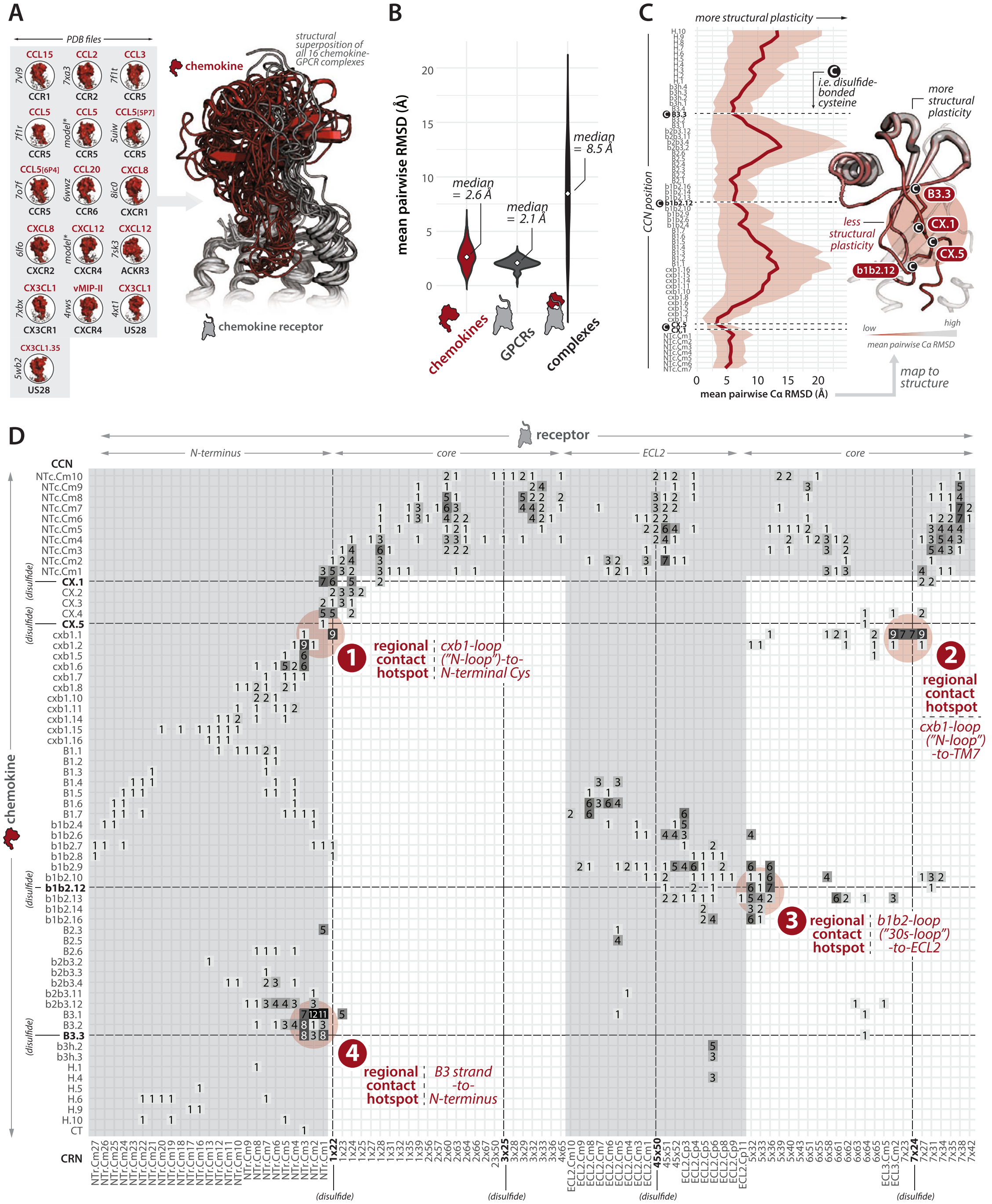

Figure S3. Structural analysis of chemokine-GPCR complexes, related to Figure 2

(A) List of 14 chemokine-GPCR complex structures and 2 models (denoted by *) used for analysis, with structure-superposition shown on right.

(B) Mean pairwise Cα RMSD after structural superposition of chemokines alone from complexes, GPCRs alone from complexes, and chemokine-GPCR complexes from (A) (STAR Methods).

(C) Mean pairwise Cα RMSD by chemokine position after structural superposition of receptors of all complexes in (A). Standard deviation is shown as shaded area. Mean pairwise Cα RMSD are plotted as "putty" on CCL3 from PDB: 7F1T with red circle highlighting area of low-RMSD positions.

(D) All chemokine-GPCR contacts from all 16 analyzed chemokine-GPCR complexes. For the chemokine, CCN positions are indicated on the left, and for the receptor, CRN positions are indicated on the bottom. Positions belonging to the N terminus of the chemokine (NTc), N terminus of the receptor (NTr), ECL2, ECL3

(legend continued on next page)

are further stratified by their position relative to conserved Cys, where "Cm1" on NTr.Cm1 indicates the position preceding the Cys (i.e., Cys minus 1), and "Cp1" in ECL2.Cp1 indicates the position following the cysteine (i.e., Cys plus 1). Boxes indicate contacts between chemokine and GPCR residue positions, with numbers indicating the number of complexes in which the residue-residue contact is present. Regional contact hotspots are numbered and labeled for reference (see Figure 2E) and highlight interface contact hotspot regions that constrain the plasticity in the chemokine-receptor-binding mode.

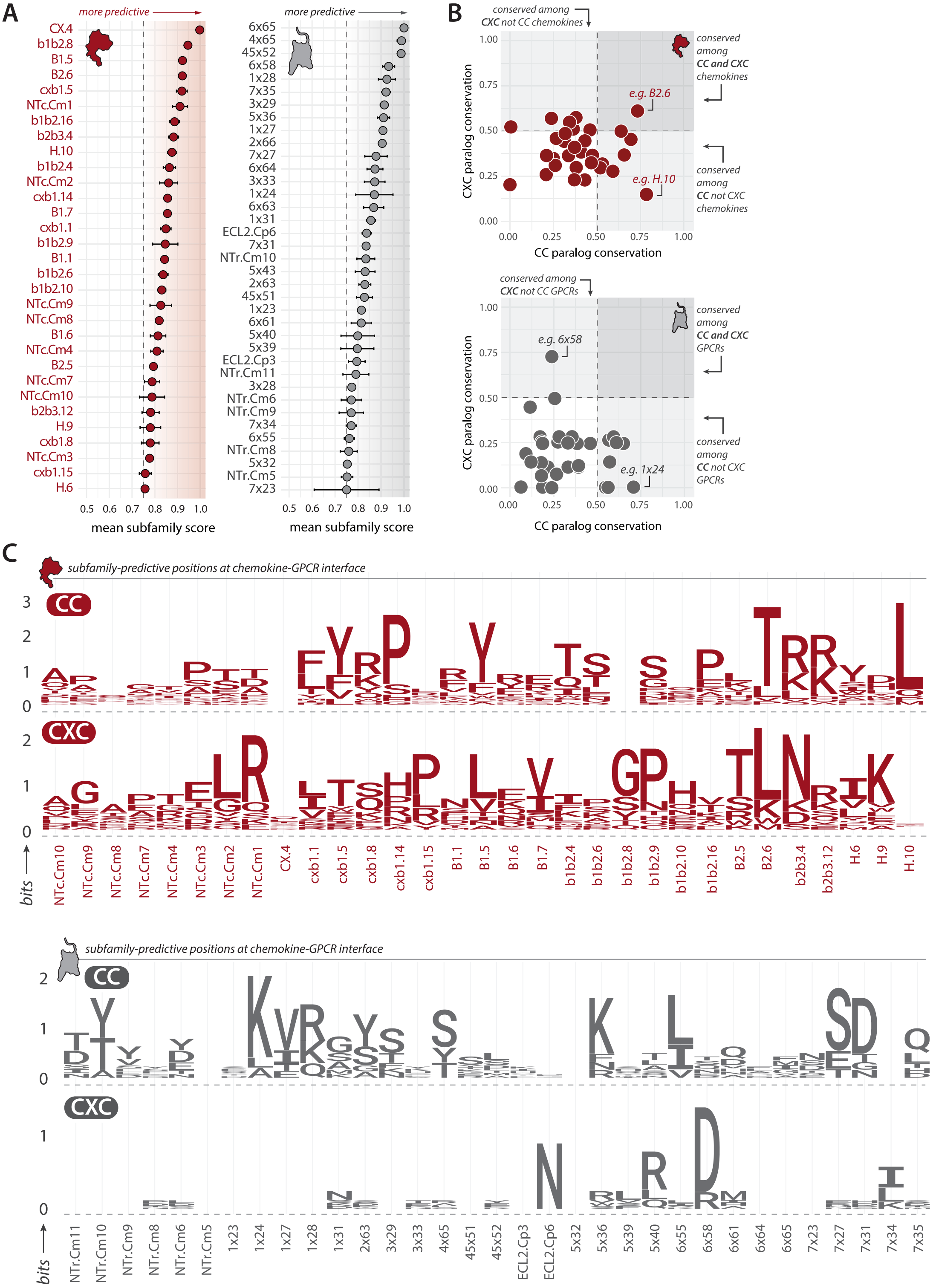

Figure S4. CC and CXC chemokine and GPCR subfamily analysis, related to Figure 3

(A) Ranked mean subfamily scores with ≥75% predictive accuracy for chemokine (left) and GPCR (right) positions making at least one contact in a chemokine-GPCR complex, with error bars reflecting SD (n = 3, STAR Methods).

(legend continued on next page)

(B) Conservation of chemokine (top) and GPCR (bottom) residue positions among CC (x axis) and CXC (y axis) human paralogs for all subfamily-predictive chemokine and GPCR positions that participate in chemokine-GPCR contacts. Contacts are only considered for human CC or CXC chemokine-GPCR complexes (i.e. PDB: 5UIW, 7O7F, 7F1R, 7VL9, 7XA3, 7F1T, 6WWZ, 6LFO, 8IC0, and models from Zheng et al.[31] and Ngo et al.[36]).

(C) Sequence logos representing amino acids among CC and CXC chemokines (top) and CC and CXC receptors (bottom) among residues that participate in interface residue contacts in human CC/CXC chemokine-GPCR complexes. Logo size scaled by bits. Absence of amino acids indicates either very low bit scores (e.g., no partial consensus) or absence of an amino acid at that alignment position.

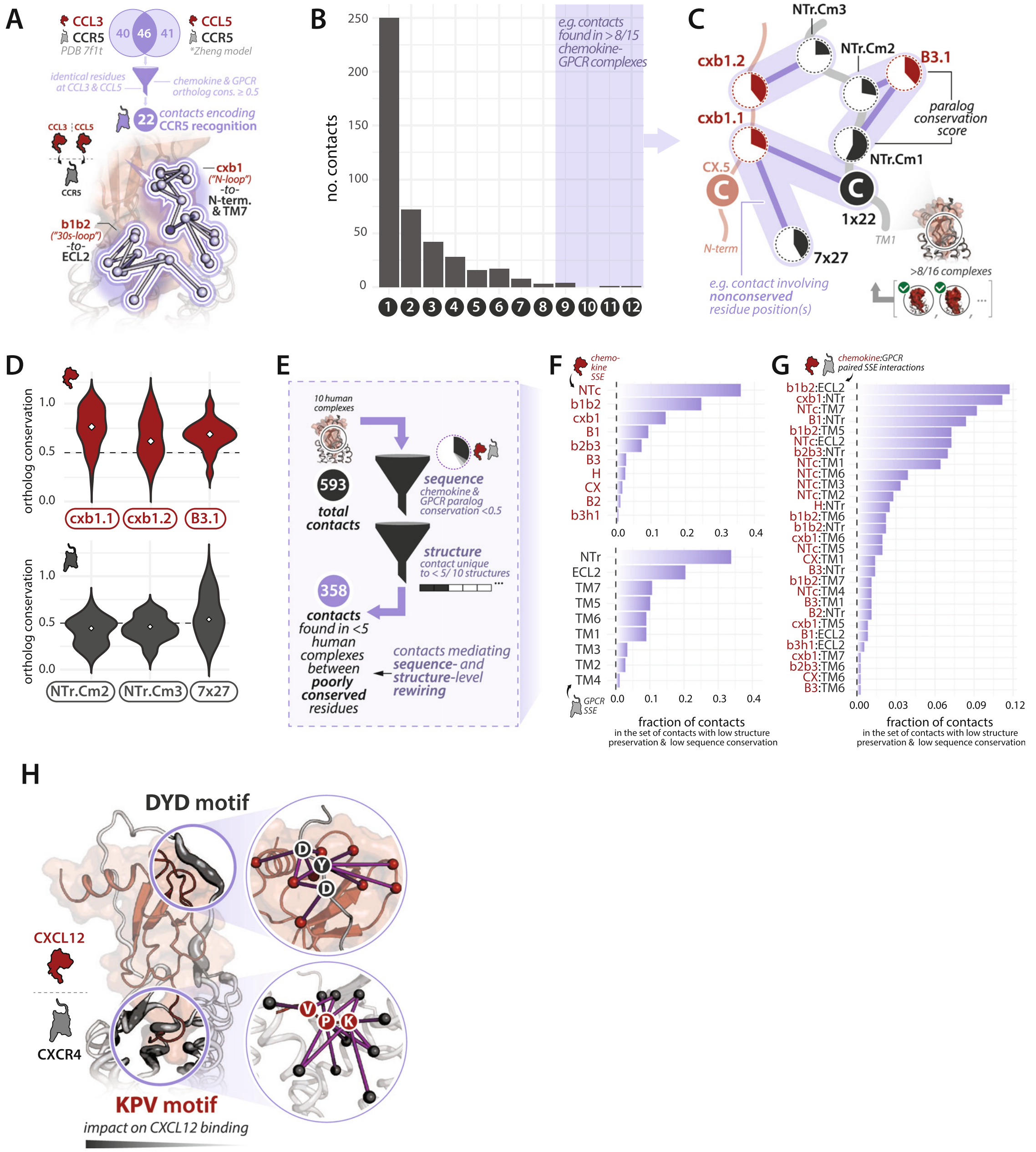

Figure S5. Sequence and structure-level changes and enrichment in chemokine- and GPCR-N termini and loops, related to Figure 4

(A) Shared residue contacts encoding CCR5 recognition were identified as contacts with the following features: (i) mediated by equivalent positions among both complexes, (ii) involved identical chemokine and GPCR residues at contacting positions among both complexes, and (iii) involved residues with ortholog conservation ≥ 0.5 among chemokine and GPCR positions.

(B) Number of contacts (y axis) shared among different numbers of chemokine-GPCR complexes (x axis). Preserved contacts are defined as those found in a majority (>8/16) chemokine-GPCR complexes.

(C) Contacts preserved in >8/16 chemokine-GPCR complexes, with pie charts representing paralog conservation of involved residues. Pie charts for receptor residues reflect paralog conservation among conventional (i.e., non-ACKR) receptors.

(D) Distribution of ortholog conservation scores for residue positions found in preserved chemokine-GPCR contacts, showing only those that have low paralog conservation (i.e., paralog conservation score < 0.5) in chemokine (top) and GPCR (bottom). Distributions include scores from 43/46 chemokines for which 1:1 ortholog alignments could be constructed and all conventional (i.e., non-atypical) chemokine receptors. Violin plots show the distribution and median (white diamond).

(E) To identify chemokine and GPCR regions that undergo the highest levels of sequence- and structure-level changes, we gathered all human chemokine-GPCR complexes (i.e., CCL5-CCR5 [model from Zheng et al. 2018], CCL15-CCR1 [PDB: 7VL9], CCL2-CCR2 [PDB: 7XA3], CCL3-CCR5 [PDB: 7F1T], CCL20-CCR6 [PDB: 6WWZ], CXCL1-CXCR1 [PDB: 8IC0], CXCL8-CXCR2 [PDB: 6LFO], CXCL12-CXCR4 [model from Ngo et al.[36]], CXCL12-ACKR3 [PDB: 7XBX], and

(legend continued on next page)

CX3CL1-CX3CR1 [PDB: 7XBX]). From these complexes, we then identified contacts that were present in fewer than five (i.e., $\leq$4/10) complexes and possessed a paralog conservation score < 0.5. GPCR paralog scores were calculated among conventional (i.e., non-ACKR) receptors.

(F) Fraction of contacts from (E) involving indicated chemokine (top) or GPCR (bottom) SSE.

(G) Fraction of contacts from (E) involving indicated pairs of chemokine-GPCR SSEs.

(H) Effects of CXCR4 mutations on CXCL12 binding (from Heredia et al.[43]) mapped onto the CXCL12-CXCR4 model, with a larger diameter and darker gray regions depicting residues experiencing the highest impact. DYD motif (CXCR4) and KPV motif (CXCL12) anchor two of the three most impacted regions.

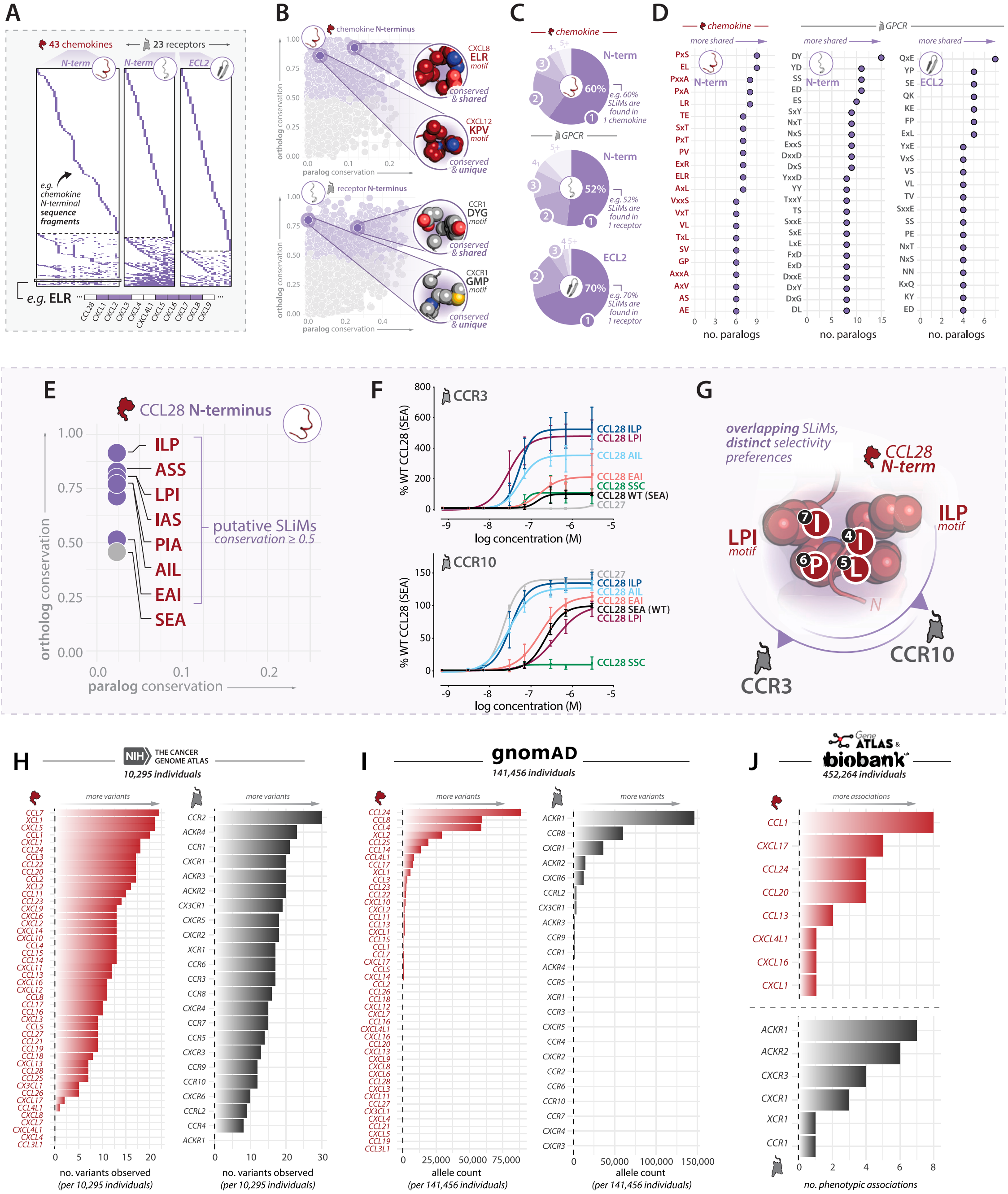

Figure S6. Identification and characterization of SLiMs, and variant identification at the chemokine-GPCR interface, related to Figure 5

(A) Sequence fragment “fingerprint” for chemokine N termini (left), receptor N termini (middle), and receptor ECL2 (right), with chemokines or receptors represented as columns and every unique fragment represented as rows. A colored, filled box indicates the presence of that fragment in a chemokine or GPCR, and a white, empty box indicates the absence of that fragment in a chemokine or GPCR. An example is shown for the chemokine ELR fragment.

(B) Ortholog and paralog conservation of all chemokine and receptor N-terminal fragments, with examples shown for putative SLiMs (defined by ortholog conservation $\geq$ 50%; purple) that are unique (e.g., KPV for CXCL12; GMP for CXCR1) or shared (e.g., ELR and DYG among multiple chemokines and receptors, respectively) among paralogous chemokines (top) and receptors (bottom). Non-SLiM fragments (ortholog conservation < 50%) are shown in gray.

(C) Pie chart representing the percentages of putative SLiMs (i.e., fragments with ortholog conservation $\geq$ 0.5) of any length that are unique to a single chemokine or receptor, shared among 2 chemokines/receptors, etc.

(D) The top, most widely shared putative SLiMs in chemokine N termini (left), receptor N termini (middle), and receptor ECL2 (right).

(E) Ortholog and paralog conservation of all 3-mer motifs in the CCL28 N terminus. Putative SLiMs (ortholog conservation $\geq$ 0.5) are shown in purple, and non-SLiM fragments (ortholog conservation < 0.5) are shown in gray.

(legend continued on next page)

(F) Calcium flux dose response of CCL28 truncation mutants at CCR3 (top) and CCR10 (bottom), normalized to CCL28 SEA (WT). Mutants are labeled according to the 3-mer peptide that initiates each mutant. All experiments $n = 3$. Error reflects SD. See Table S2.

(G) Overlapping-but-distinct CCL28 SLiMs have different roles in CCR3 and CCR10 selectivity.

(H) Number of cancer-associated variants observed for all chemokines and receptors at chemokine-GPCR interface positions from The Cancer Genome Atlas (TCGA).

(I) The observed allele counts for all chemokines and receptors at chemokine-GPCR interface positions from the gnomAD database.

(J) The number of unique phenotypic associations per gene among phenotype-associated variants occurring at chemokine-GPCR interface positions with p value $< 1e{-8}$ for chemokines (top) and receptors (bottom).

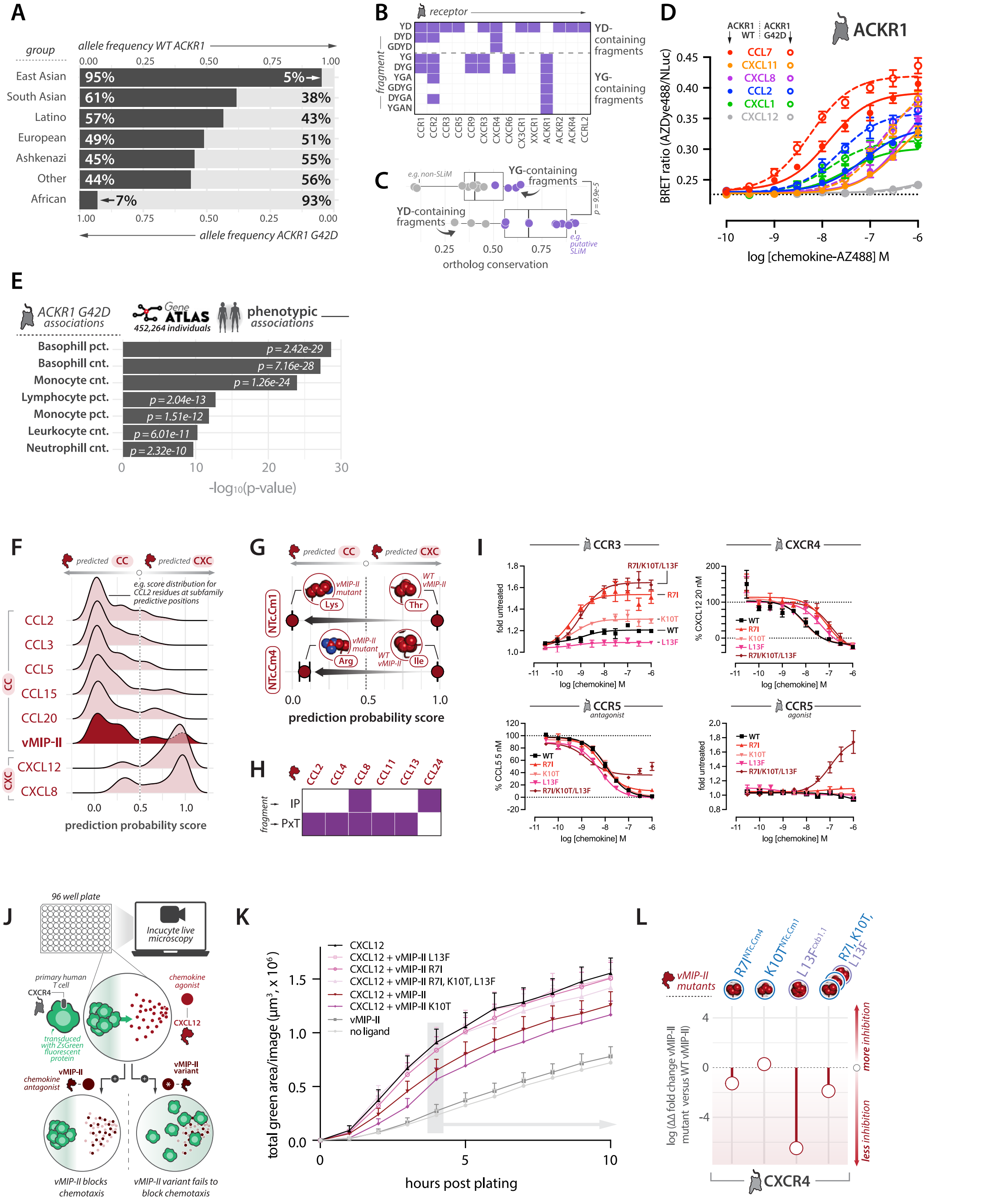

Figure S7. Characterization of a naturally occurring variant of ACKR1 in the N terminus and design and testing of a re-engineered viral chemokine, related to Figures 5 and 6

(A) Allele frequency among different groups was calculated from allele counts and numbers in the gnomAD data for ACKR1 G42 (WT; dark gray) versus D42 (light gray).

(B) Comparison of YD- and YG-containing fragments that are present in ACKR1 across chemokine receptors.

(C) Ortholog conservation of YG- and YD-containing fragments (excluding gapped fragments) that are present among chemokine receptors. ACKR1 fragments themselves were excluded to selectively assess conservation and infer the function of these fragments more broadly among the chemokine-receptor family. The *p* value of the difference is from the Wilcoxon test. Boxplot parameters as in Figure 1H.

(legend continued on next page)

(D) Dose-response BRET-based binding assay with AzDye488-labeled chemokines and NLuc-labeled WT or G42D ACKR1. All conditions n = 3. See Table S2.
(E) Phenotypic associations of ACKR1 G42D from GeneAtlas with p values reported in Canela-Xandri et al.[54]
(F) Histograms for a series of chemokines representing prediction probability scores for all residues at positions that are predictive of the CC versus CXC subfamily (subfamily score $\geq$ 0.75) (STAR Methods). This contrasts with Figure 6B which reflects only interface positions. Scores assess the likelihood that a queried residue belongs to a CC (i.e., closer to 0) or CXC (i.e., closer to 1) chemokine.
(G) Changes in prediction probability score as a consequence of mutating the indicated residue in WT vMIP-II.
(H) Distribution of IP and PxT fragments among CCR3- and/or CCR5-binding chemokines. A purple square indicates that the fragment is present in the respective chemokine. All fragments are putative SLiMs (i.e., conservation $\geq$ 0.5) apart from PxT in CCL11 (conservation score = 0.39).
(I) β-arrestin-1 recruitment assays via NanoBiT. All assays n = 3. Error bars reflect SEM. CXCR4 was investigated in antagonist mode with co-treatment with 20 nM CXCL12 (agonist) and varying concentrations of vMIP-II mutant (x axis) in dose response. CCR5 was investigated in both antagonist (bottom left) and agonist (bottom right) modes. In antagonist mode, CCR5 was co-treated with 5 nM CCL5 (agonist) and tested against varying concentrations of vMIP-II mutant (x axis) in dose response. See Table S2.
(J) Schematic demonstrating chemotaxis assay setup.
(K) Chemotaxis assay with purified, CXCR4-expressing, fluorescently labeled human T cells in response to CXCL12 alone, vMIP-II alone, CXCL12 (50 ng/mL) with vMIP-II mutants, or in the absence of ligand. Error bars reflect SEM.
(L) The log ratio of the difference between CXCL12 migration area and CXCL12/vMIP-II mutant (numerator) and the difference between CXCL12 migration area and CXCL12/WT vMIP-II (denominator) for indicated vMIP-II mutants. Positive deflections represent diminished chemotaxis compared with WT vMIP-II (i.e., more pronounced inhibition of CXCL12-stimulated chemotaxis), whereas negative deflections represent enhanced chemotaxis compared with WT vMIP-II (i.e., less pronounced inhibition of CXCL12-stimulated chemotaxis).
